# The apoptotic machinery as a biological complex system: analysis of its omics and evolution, identification of candidate genes for fourteen major types of cancer, and experimental validation in CML and neuroblastoma

**DOI:** 10.1186/1755-8794-2-20

**Published:** 2009-04-30

**Authors:** Cinzia Di Pietro, Marco Ragusa, Davide Barbagallo, Laura R Duro, Maria R Guglielmino, Alessandra Majorana, Rosario Angelica, Marina Scalia, Luisa Statello, Loredana Salito, Luisa Tomasello, Salvo Pernagallo, Salvo Valenti, Vito D'Agostino, Patrizio Triberio, Igor Tandurella, Giuseppe A Palumbo, Piera La Cava, Viviana Cafiso, Taschia Bertuccio, Maria Santagati, Giovanni Li Destri, Salvatore Lanzafame, Francesco Di Raimondo, Stefania Stefani, Bud Mishra, Michele Purrello

**Affiliations:** 1 Dipartimento di Scienze BioMediche, Sezione di Biologia Generale, Biologia Cellulare, Genetica Molecolare *G Sichel*, Unità di Biologia Genomica e dei Sistemi Complessi, Genetica, Bioinformatica, Università di Catania, 95123 Catania, Italy; 2 Dipartimento di Scienze BioMediche, Sezione di Ematologia, Università di Catania, 95125 Catania, Italy; 3 Dipartimento di Scienze Ginecologiche e Microbiologiche, Università di Catania, 95125 Catania, Italy; 4 Dipartimento di Scienze Chirurgiche, Università di Catania, 95125 Catania, Italy; 5 Dipartimento di Anatomia, Patologia Diagnostica, Medicina Legale, Igiene e Sanità Pubblica, Università di Catania, 95125 Catania, Italy; 6 Courant Institute of Mathematical Sciences, New York University, New York, NY 10012, USA

## Abstract

**Background:**

Apoptosis is a critical biological phenomenon, executed under the guidance of the Apoptotic Machinery (AM), which allows the physiologic elimination of terminally differentiated, senescent or diseased cells. Because of its relevance to BioMedicine, we have sought to obtain a detailed characterization of AM Omics in *Homo sapiens*, namely its Genomics and Evolution, Transcriptomics, Proteomics, Interactomics, Oncogenomics, and Pharmacogenomics.

**Methods:**

This project exploited the methodology commonly used in Computational Biology (i.e., mining of many *omics *databases of the web) as well as the High Throughput biomolecular analytical techniques.

**Results:**

In *Homo sapiens *AM is comprised of 342 protein-encoding genes (possessing either anti- or pro-apoptotic activity, or a regulatory function) and 110 MIR-encoding genes targeting them: some have a critical role within the system (*core *AM nodes), others perform tissue-, pathway-, or disease-specific functions (*peripheral *AM nodes). By overlapping the cancer type-specific AM mutation map in the fourteen most frequent cancers in western societies (breast, colon, kidney, leukaemia, liver, lung, neuroblastoma, ovary, pancreas, prostate, skin, stomach, thyroid, and uterus) to their transcriptome, proteome and interactome in the same tumour type, we have identified the most prominent AM molecular alterations within each class. The comparison of the fourteen mutated AM networks (both protein- as MIR-based) has allowed us to pinpoint the hubs with a general and critical role in tumour development and, conversely, in cell physiology: in particular, we found that some of these had already been used as targets for pharmacological anticancer therapy. For a better understanding of the relationship between AM molecular alterations and pharmacological induction of apoptosis in cancer, we examined the expression of AM genes in K562 and SH-SY5Y after anticancer treatment.

**Conclusion:**

We believe that our data on the Apoptotic Machinery will lead to the identification of new cancer genes and to the discovery of new biomarkers, which could then be used to profile cancers for diagnostic purposes and to pinpoint new targets for pharmacological therapy. This approach could pave the way for future studies and applications in molecular and clinical Medicine with important perspectives both for Oncology as for Regenerative Medicine.

## Background

Cells use sophisticated mechanisms to functionally connect their molecules and machineries with the aim of activating, sustaining and modulating their critical functions: survival, growth, proliferation, differentiation, and death [1,2]. Following the characterization of very complex cross-talks among the different signalling cascades, a molecular network view of cell biology and physiology has emerged together with the concept of Biological Complex Systems [3]. The ultimate outcome of this structural and functional organization is the metabolism of organisms and their cells. Recently, the striking evolution of experimental HT (High Throughput) strategies (i.e., computational, molecular, cellular, and systemic techniques) has made it possible for Biology to become holistic, thus providing also a top > down view of organisms [4]. The complex biological system *par excellence *is the human being. However, because of the technological and conceptual limits that this type of study still presents, it seems appropriate to focus on more discrete entities such as the molecular machineries specifically responsible for a phenotypic phenomenon or a biomolecular function (e.g., the Transcription Apparatus, TA, or the Apoptotic Machinery, AM) [5,6]. The critical importance of apoptosis for the BioPathology of *Homo sapiens *is stressed by the following considerations. Apoptosis performs a central role during development and differentiation (e.g., morphogenesis, immune and nervous system development, and sexual differentiation), and at steady state during adult life (e.g., tissue homeostasis, elimination of damaged or abnormal cells, and defence against infections) [7,8]: for instance, each day about one in a million of our cells dies (about 50–100 × 10^9 ^out of 50–100 × 10^15^), mostly via apoptosis, to be replaced through stem cells proliferation and differentiation [9]. Unsurprisingly, apoptosis is the most common and evolutionarily conserved among the mechanisms causing cell death [10,11]. Its critical biological functions make it very likely that AM dysfunctions also would have an important pathogenetic role in many diseases [12,13]. It is known that an abnormal increase of apoptosis is involved in degenerative diseases (e.g., Diabetes, Arterioschlerosis), neurodegenerative diseases (e.g., Alzheimer's and Parkinson's Disease), autoimmune diseases (e.g., Multiple Sclerosis), ischemic pathologies (e.g., myocardial infarction), pathologies caused by toxins (e.g., hepatitis induced by alcohol), viral or bacterial infections (e.g., HIV or *Neisseria meningitidis*) [14-16]. On the other hand, inhibition of apoptosis is present in most cancers: in fact, one of the critical goals of contemporary Oncology is to understand how cancer cells evade death, both the one endogenously activated as well as that induced by host mechanisms of immune surveillance or by therapeutic treatments [17-19]. Accordingly, molecular profiling of apoptotic pathways within the AM of a specific cancer should be critical to rationally design strategies toward its elimination [20,21]. Incidentally, this work may lead to understanding the molecular bases of cancer *immortality *[22,23]. In this paper, we focus on AM *Omics *in *Homo sapiens *(Genomics, Transcriptomics including MIRs, Proteomics including PTMs and NUPs, Interactomics, Oncogenomics, and Pharmacogenomics) and describe AM evolution through the analysis of its structure in ninety organisms from *Homo sapiens *to *Escherichia coli*. We then use these data to characterize AM Omics in the fourteen most frequent cancer types in the Western World, with the aim of identifying new markers for the design of innovative antineoplastic strategies. Finally, we experimentally validate our work through HT analysis of AM transcriptome changes in CML and neuroblastoma, after *in vitro *treatment with commonly used therapeutic drugs.

## Methods

### Dataset of genes involved in apoptosis

Because of the intrinsic nature of biological systems and processes, the unequivocal identification of a gene's involvement in a specific process is nontrivial. Since genes participate in a varied spectrum of biomolecular phenomena with a varying degree of involvement, the experimental work involves inference that admits some false positives and negatives and hence does not always necessarily yield clear-cut answers. Nonetheless, within a cellular machinery such as AM it is possible to identify: (i) putative *core *nodes, ubiquitously expressed genes endowed with a central biomolecular role in the activation or inhibition of apoptosis (e.g., death receptors, members of the Bcl2 family, caspases); (ii) putative *peripheral *nodes, genes with tissue-, pathway-, or disease-specific functions: in many cases these perform a regulatory role. We first searched the GO database [24] by using as key words *apoptosis *and *cell death *and compiled a preliminary list of human genes associated with apoptosis; this was then manually filtered by using literature data from PubMed, in order to experimentally verify the involvement of these genes in apoptosis [25]. The final list of AM genes was compiled by using interactome data for verifying the functional connections among these proteins [26,27]. This approach (i.e., combining GO, PubMed and Interactome data) allowed us to establish a dataset consisting of 342 genes involved in apoptosis, whose products were physically or functionally interacting. The experimentally supported data of interactions between AM transcripts and MIRs were extracted from Tarbase [28]. Recent comparative studies among target prediction methods have shown that no single one of them is significantly better than others. Accordingly, it has become common to use multiple prediction tools, concentrating on the intersecting results, in order to overcome the well-known problems related to MIR targets identification [29]. In this project, we used miRGen [30], which intersects the results from three different classic tools (miRanda, PicTar, and TargetScan) to predict the MIRs controlling AM genes. The data about MIRs genomic position were extracted from miRBase [31].

### Genomics

The cytogenetic and genomic position of each AM gene was obtained by searching the database *Gene *of NCBI [25]. The genome map of AM genes was displayed by *Caryoscope *[32] to identify the position of AM genes in the genome. The distribution of AM genes in each chromosome was compared to that of all human genes [33]. AM cytogenetic map was compared to that of tumour-associated chromosomal structural mutations. The latter was determined by using data from the Mitelman database and CGH informations from Progenetix (a database of cytogenetic abnormalities in cancer) for the most frequent anatomic cancer sites (breast, cervix, colon, kidney, liver, lung, neuroblastoma, ovary, pancreas, prostate, skin, stomach, and thyroid) [34,35]. The overlap of the two maps was examined through *Caryoscope*. A red-green matrix (AM genes × tumours, in which the positive values indicate the frequency of gains while the negative ones indicate the frequency of losses) led to identification of AM genes clusters: these were displayed with MeV4.2 [36]. The information on cancer-related gene mutations was obtained by screening the database Cosmic [37]. Cancer-related methylation data were from PubMeth [38].

### Evolution

To characterize the phylogenetic distribution of AM proteins, we collected AM orthologs from a large number of *taxa *[Prokaryotes (*Bacteria*, *Archaea*), Unicellular Eukaryotes (*Alveolata*, *Diplomonadida *group, *Entamoebidae*, *Euglenozoa*, *Mycetozoa*), *Viridiplantae*, *Fungi*, Invertebrates (*Acoelomata*, *Pseudocoelomata*, *Protostomia*), Vertebrates (*Actinopterygii*, *Amphibia*, *Sauropsida*, *Mammalia*)] and Viruses (both dsDNA viruses and retro-transcribing viruses) from the databases Homologene [39], iProClass [40] and Metazome [41]. The search for AM orthologs was performed by using BLASTp with human AM proteins as queries against all protein databases [42]. The total number of organisms used for analyzing AM phylogenesis was ninety. The evolutionary rate of AM proteins was assessed by carrying out a multiple alignment (MA) of each human protein with its orthologs from a series of model organisms (*Mus musculus*, *Gallus gallus*, *Xenopus laevis*, *Danio rerio*, *Takifugu rubripes*, *Drosophila melanogaster*, *Caenorhabditis elegans*, *Dictyostelium discoideum*, *Bacillus subtilis*, *Methanococcus jannaschii*). MA was performed with ClustalW [43], while aligned sequences were processed with Mega 3.0 [44] to obtain the Poisson Corrected distance (k): this provides an estimate of the difference between two sequences (meant as percentage of amino acid substitutions) for all pairs of orthologs. The evolutionary rate (ν) was calculated through Kimura's parameter, ν = k/2 t, where t represents the time of divergence between two species [45]. *Histone H4 *(a highly conserved protein), SOD2 (a protein with an average degree of conservation) and *Fibrinopeptide *(a protein characterized by a low conservation level) were used as evolutionary markers [46]. The evolutionary rate was calculated for those proteins expressed at least in all vertebrates.

### Transcriptomics

Gene expression data on AM genes were obtained from three datasets: Human Transcriptome Map (HTM), NCI60 Cancer Microarray Project, and Oncogenomics for cancer and normal tissues [47-49]. Because of the small sample size, fdr (false discovery rate) analysis with an empirical null model (as proposed by Efron) was deemed infeasible. Instead, the t-test was applied to identify those genes with expression values that differ significantly between normal and tumour samples (for HTM and Oncogenomics datasets) in each cancer model. We performed a test between subjects: Group A (normal tissues), Group B (cancer tissues); Welch approximation; Alpha (overall threshold p-value) = 0.001; the p-value is based on permutation (100 permutation per gene); the significance was determined by the Adjusted Bonferroni Correction. The resulting differentially expressed genes were further analyzed: for each differentially expressed gene, in each cancer model, we calculated the natural logarithm of the ratio between the mean of expression values of group B and the mean of expression values of group A. We chose the stringent cut-off of +1 and -1 (about three fold up- or down-regulated) to determine the up regulation and down regulation, respectively: the genes with *ln *ratio values between +1 and -1 were considered not significantly changed [50,51]. To further strengthen our analysis, we compared and completed these data with expression values from NCI60 datasets (analyzed as described above). Data analysis was performed and displayed with MeV4.2. The overlapping of AM transcriptome map with the AM mutation map was performed by Caryoscope. Expression data on MIRs, that target AM genes in different normal and tumour tissues, were derived from the database VITA [52], which contains MIR profiling obtained from bead-based flow cytometry of mature forms of MIRs [53].

### Proteomics

AM protein expression data were obtained from The Human Protein Atlas and NCI60 protein datasets [54,55]. Due to the incomplete structure of these databases, some important cancer-related proteins were not included in our analysis. The data concerning protein features such as PTM, the presence of metal ions or an intrinsically unfolded structure, were obtained from HPRD and DisProt databases [27,56]. The structural characterization of protein motifs and domains was performed by screening the database ExPASy [57].

### Interactomics

Data on protein-protein and protein-DNA interactions were obtained from BIND [26] and HPRD [27] databases, while pathway data are from Kegg and Biocarta [58,59]. The AM interactome was analyzed and visualized by Cytoscape [60], CentiBin [61], and iVici [62]. By using Cytoscape plug-ins, interactome data were interpolated with structural information on AM proteins, to derive the relationships between structural motifs and interactions. Phenome data from knock-out experiments in the mouse were obtained from Mouse Genome Informatics [63]. Drug targeting information was extracted from DrugBank [64].

### Cell lines and culture conditions

The K562 cell line was derived from a patient with CML in blast crisis and expressing the chimaeric non-receptor tyrosine kinase p210^Bcr-Abl ^[65]. Cell were cultured in RPMI 1640-glutamax, supplemented with 10% heat-inactivated fetal bovine serum (FBS; Gibco BRL), 2% L-glutamine, 1% penicillin/streptomycin. Cells were grown under 5% CO_2_/95% O_2 _in a 37°C humified incubator. Imatinib (STI571, Gleevec) was provided by Novartis Pharma (Basel, Switzerland). A 10 mM stock solution was freshly prepared in sterile phosphate-buffered saline and diluted in RPMI 1640 medium before use. K562 cells, exposed to 1 μM imatinib and relative controls, were collected and analyzed at different time points (0 h, 2 h, 4 h, 7 h, 12 h, 24 h, 48 h, 72 h). Human SH-SY5Y neuroblastoma cells, derived from a patient with a bone marrow metastasis [66], were grown in a 1:1 mixture of DMEM and Ham's F-12 medium (Cambrex Bio Science, Verviers Belgium), supplemented with 10% fetal bovine serum, 1% L-glutamine and 1% penicillin/streptomycin (Gibco, Invitrogen, Carlsbad, CA), in a humidified atmosphere of 5% CO_2 _in air. For apoptosis induction, 3 × 10^6 ^cells were seeded into 75 cm^2 ^flasks in 15 ml of culture medium. Fenretinide (dissolved in absolute ethanol) was added to cultures to a final concentration of 3 μM; an equal volume of ethanol (~0.3% of culture volume) was used to treat control cells. SH-SY5Y cells exposed to 3 μM fenretinide and relative controls were collected and analyzed at different time points (0 h, 12 h, 24 h, 48 h, 72 h, 96 h).

### Cell population dynamics and detection of apoptosis or necrosis

To detect apoptosis or necrosis, one aliquot (2 × 10^5 ^cells) was used for flow cytometric analysis. Cell fluorescence was assessed with a Facscalibur flow cytometer (Becton Dickinson, San Jose, CA) by using the CellQuest software (Becton Dickinson). Standard protocols were followed to determine annexin-positivity with an Annexin V-FITC Apoptosis Detection Kit (Sigma, Saint Louis, Missouri).

### Real – time PCR

Total RNA was extracted with Trizol (Invitrogen) from treated cells and untreated controls after trypsinization (for the neuroblastoma cell line) and centrifugation (for both lines) at 1200 rpm for 10 min. Total RNA (3 μg) was reverse-transcribed using SuperScript II and random hexamers (Roche Diagnostics GmbH, Mannheim, Germany) [19]. cDNA (30 ng) was added to each well of a PCR array for quantitative PCR (Apoptosis PCR arrays and RT^2 ^Profiler PCR Array, SuperArray Bioscience Corporation, MD, USA). The array consisted of 96 primers for 84 genes of AM *core *protein-encoding genes, plus five housekeeping genes and three RNA and PCR quality controls. PCR cycles were performed according to the manufacturer instructions. The relative level of mRNA expression for each gene in each sample was first normalized to the expression of housekeeping genes (also provided in the array) in treated samples and then normalized with respect to the level of cDNA expression in control samples according to the 2^-ΔΔ*CT *^method [67]. Quantitative real-time PCR was performed on Mx3005P™ QPCR system (Stratagene, La Jolla, CA, USA). The results were considered significant when the expression of a specific cDNA was at least either three times higher or lower than that of controls.

## Results

### Physiology

#### AM general features

The 342 protein-encoding genes, that we assigned to *Homo sapiens *AM, encode 596 different mRNAs that are translated into 548 proteins (Tables 1, 2; Additional files 1, 2): 25% of them are involved in apoptosis induction, 20% in its inhibition, 40% in regulation, 15% in tissue-, pathway-, and disease-specific functions. Despite their common involvement with apoptosis, AM proteins are highly heterogeneous according to three GO categories: (i) Biological Process; (ii) Molecular Function; (iii) Subcellular Location (Additional file 2). As expected, the most represented biological process is apoptosis (65%), but many also are involved in metabolism (54%) or cell communication (55%) (Additional file 2). The most frequent molecular functions of AM proteins are receptor- or kinase-activity within signal transduction pathways (20% each), nucleic acid binding (17%), transcription factor activity (9%) (Additional file 2); their most frequent subcellular localizations are the nucleus (51%), the plasma membrane (43%), and mitochondria (10%) (Additional file 2). We identified 110 MIRs whose targets are AM protein-encoding genes, of which fourteen had been experimentally validated (Additional files 1 and 3). We also found other five MIR-encoding genes physically located within introns of AM protein-encoding genes: while waiting for the experimental verification of their involvement in AM functions, we propose to temporarily assign them to the AM (Additional file 4). Accordingly, the total count of AM genes would amount to 457. Interestingly, MIRs of the Apoptotic Machinery preferentially target AM protein-encoding genes that negatively regulate apoptosis or control cell cycle through transcription factors and protein serine/threonine kinases (Additional file 2).

**Table 1:** Pro AM core genes

**AM GENES**	**FAMILY**	**PATHWAY**	**FUNCTION**
APAF1		p53 Pathway, Apoptosome	PRO

BAD		Apoptotic Mitochondrial Pathway	PRO

BAK1	BCL-2 RELATED	Apoptotic Mitochondrial Pathway	PRO

BAX	BCL-2 RELATED	Apoptotic Mitochondrial Pathway, p53 Pathway	PRO

BCL2L1	BCL-2 RELATED	Apoptotic Mitochondrial Pathway	PRO*

BID	BCL-2 RELATED	Apoptotic Mitochondrial Pathway	PRO

CARD12	NALP	Caspase Cascade	PRO

CARD4	NALP	Caspase Cascade	PRO

CARD8	NALP	Caspase Cascade	PRO

CASP10	CASPASE	Caspase Cascade	PRO

CASP3	CASPASE	Caspase Cascade	PRO

CASP6	CASPASE	Caspase Cascade	PRO

CASP7	CASPASE	Caspase Cascade	PRO

CASP8	CASPASE	Caspase Cascade	PRO

CASP9	CASPASE	Caspase Cascade, Apoptosome	PRO

CFLAR	CASPASE	FAS Signaling Pathway	PRO*

CHUK	SER/THR KINASE	AKT Signaling Pathway, Induction of Apoptosis through DR3 and DR4/5 Death Receptors	PRO

CYCS		Caspase Cascade, Apoptosome	PRO

DAP		IFN-Gamma-Induced Cell Death	PRO

DAP3		IFN-Gamma-Induced Cell Death, FAS Signaling Pathway, SODD/TNFR1 Signaling Pathway	PRO

DAPK1		IFN-Gamma-Induced Cell Death	PRO

DAXX		FAS Signaling Pathway	PRO

DFFA		Apoptotic DNA Fragmentation and Tissue Homeostasis	PRO

DFFB		Apoptotic DNA Fragmentation and Tissue Homeostasis	PRO

FADD		FAS Signaling Pathway	PRO

FAS	TNF RECEPTOR	FAS Signaling Pathway	PRO*

FASLG	TNF	FAS Signaling Pathway	PRO

HRK		Apoptotic Mitochondrial Pathway	PRO

IL1A	INTERLEUKIN 1 CYTOKINE	NF-kB Signaling Pathway	PRO*

IL1R1	INTERLEUKIN 1 RECEPTOR	NF-kB Signaling Pathway	PRO*

MCL1	BCL-2 RELATED	Apoptotic Mitochondrial Pathway	PRO*

NALP1	NALP	FAS Signaling Pathway	PRO

NFKBIA		AKT Signaling Pathway, Induction of Apoptosis through DR3 and DR4/5 Death Receptors, NF-kB Signaling Pathway	PRO

RIPK1	SER/THR KINASE	NF-kB Signaling Pathway, Induction of Apoptosis through DR3 and DR4/5 Death Receptors	PRO

TNF	TNF	SODD/TNFR1 Signaling Pathway	PRO*

TNFRSF10A	TNF RECEPTOR	Induction of Apoptosis through DR3 and DR4/5 Death Receptors, Natural Killer Cell Mediated Cytotoxicity	PRO

TNFRSF10B	TNF RECEPTOR	p53 Pathway, Natural Killer Cell Mediated Cytotoxicity	PRO

TNFRSF1A	TNF RECEPTOR	SODD/TNFR1 Signaling Pathway	PRO

TNFSF10	TNF	Induction of Apoptosis through DR3 and DR4/5 Death Receptors	PRO

TP53	TP53-RELATED	Apoptotic Signaling in Response to DNA Damage, ATM Signaling Pathway,	PRO

TRADD		Induction of Apoptosis through DR3 and DR4/5 Death Receptors	PRO

TRAF2	TRAF	Induction of Apoptosis through DR3 and DR4/5 Death Receptors	PRO*

The symbol "*" means involvement also in anti-apoptotic functions.

**Table 2:** Anti AM core genes

**AM GENES**	**FAMILY**	**PATHWAY**	**FUNCTION**
AKT1	SER/THR KINASE	FAS Sgnaling Pathway	ANTI

BAG1	BCL2-ASSOCIATED ATHANOGENE	SODD/TNFR1 Signaling Pathway	ANTI

BAG3	BCL2-ASSOCIATED ATHANOGENE	SODD/TNFR1 Signaling Pathway	ANTI

BAG4	BCL2-ASSOCIATED ATHANOGENE	SODD/TNFR1 Signaling Pathway	ANTI

BCL2	BCL-2 RELATED	Apoptotic Mitochondrial Pathway, p53 Pathway	ANTI

BCL2L1	BCL-2 RELATED	Apoptotic Mitochondrial Pathway	ANTI*

BIRC2	BIRC	Apoptotic Mitochondrial Pathway, Caspase Cascade	ANTI

BIRC3	BIRC	Apoptotic Mitochondrial Pathway, Caspase Cascade, SODD/TNFR1 Signaling Pathway	ANTI

BIRC4	BIRC	Apoptotic Mitochondrial Pathway, Caspase Cascade, B Cell Survival Pathway	ANTI

BIRC5	BIRC	B Cell Survival Pathway	ANTI

CFLAR	CASPASE	FAS Signaling Pathway	ANTI*

FAS	TNF RECEPTOR	FAS Signaling Pathway	ANTI*

IL1A	INTERLEUKIN 1 CYTOKINE	NF-kB Signaling Pathway	ANTI*

IL1R1	INTERLEUKIN 1 RECEPTOR	NF-kB Signaling Pathway	ANTI*

MAP3K14	SER/THR KINASE	NF-kB Signaling Pathway, Induction of Apoptosis through DR3 and DR4/5 Death Receptors	ANTI

MCL1	BCL-2 RELATED	Apoptotic Mitochondrial Pathway	ANTI*

MYD88		NF-kB Signaling Pathway	ANTI

NFKB1		AKT Signaling Pathway, Induction of Apoptosis through DR3 and DR4/5 Death Receptors, NF-kB Signaling Pathway	ANTI

NFKB2		AKT Signaling Pathway, Induction of Apoptosis through DR3 and DR4/5 Death Receptors, NF-kB Signaling Pathway	ANTI

RELA		NF-kB Signaling Pathway	ANTI

TNF	TNF	SODD/TNFR1 Signaling Pathway	ANTI*

TNFRSF10C	TNF RECEPTOR	Natural Killer Cell Mediated Cytotoxicity	ANTI

TNFRSF10D	TNF RECEPTOR	Natural Killer Cell Mediated Cytotoxicity	ANTI

TRAF2	TRAF	Induction of Apoptosis through DR3 and DR4/5 Death Receptors	ANTI*

The symbol "*" means involvement also in pro-apoptotic functions.

#### AM genomics and evolution

**Figure 1:**
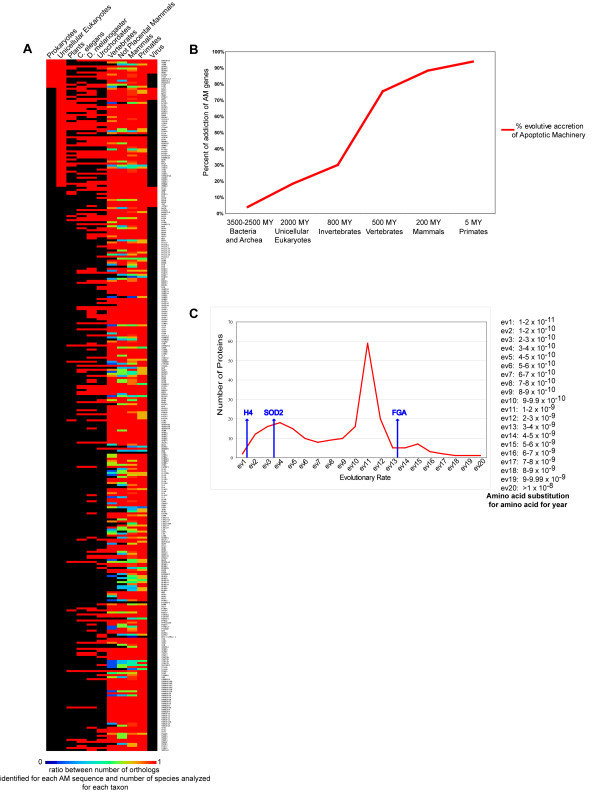
**AM evolution**. Panel A. Matrix of phylogenetic distribution of AM genes in all *taxa*. Each row represents the color coded ratio between ortholog number for individual AM genes and species analyzed in a given *taxon*, according to the colour bar shown above the matrix; the columns represent the *taxa*. Panel B. Evolutionary accretion of AM: number of AM genes appeared in the genomes during evolution. Panel C. Evolutionary rate of AM proteins: the X-axis represents the distribution classes of evolutionary rates, indicated as amino acid substitution for 100 amino acids per year as shown in the caption to the right. The Y-axis represents the number of proteins in each class of evolutionary rate.

AM genes are uniformly spread in the human genome, including the pseudoautosomal regions of X and Y chromosomes (Additional file 5). However, we also identified small clusters of phylogenetically related sequences: five genes of the TNFR family and two DNA fragmentation factors are localized at 1p36; three interferon genes map at 9p21; two members of the BIRC family and four caspases are at 11q22; three members of the STAT family are localized at 17q21; four members of the NALP family are localized at 19q13; two Interferon Receptors are at 21q22 (Additional file 6). These clusters are conserved in at least two other mammals (*Pan troglodytes *and *Mus musculus*) with the exception of CASP5 and NALP8, not found in the mouse, and the CASP12 pseudogene that turns out to be an expressed gene in both the chimpanzee and the mouse (Additional file 6). We also detected MIR-encoding genes physically located within introns or UTRs of AM protein-encoding genes; it is at present unknown if these MIRs are involved in AM functions (Additional file 4). Phylogenetic analysis of unicellular eukaryotes demonstrated the presence of several orthologs of human AM genes, for instance *Programmed Cell Death 5 and 6 *(PDCD5 and PDCD6) that are involved in apoptosis-like phenomena (Figure 1, Panel A). About 4% of human AM genes, belonging to different GO categories (transcription factors, kinases, regulators of the cell cycle or of proliferation, differentiation, and apoptosis), appear to have orthologs in *Bacteria *and *Archaea*; however, these genes are *peripheral *rather than *core *AM nodes (Figure 1, Panel A). Cytochrome c is the only gene with a central role in apoptosis, found in the Domains of both *Prokarya *and *Archaea*; in metazoans the protein is a constitutive member of the apoptosome, whilst in more ancestral organisms it is mainly involved in oxidative phosphorylation (Figure 1, Panel A). About 7% of human AM genes are found inside viral genomes (both DNA and RNA viruses): they encode transcription factors involved in cell cycle control, proliferation, and differentiation (Figure 1, Panel A). Our phylogenetic analysis of AM structure in a large series of organisms confirmed the evolutionary increase of the number of AM genes, concurrent with that of AM structure complexity (Figure 1, Panel B). AM functional core (BCL2 family members, Death Associated Proteins, BAG, BIRC, Caspases) appeared in multicellular organisms and was enriched with new molecular elements (e.g., DEATH receptors and their ligands, CARDs and TRAFs) with the onset of vertebrates. The emergence of mammals coincided with a further increase in the number of AM genes: this presumably was caused by the expansion of gene families that characterized the evolution of these genomes (Figure 1, Panel B). Analysis of the evolutionary rate (*v*) of AM proteins has allowed us to establish the evolutionary trend of AM (Figure 1, Panel C). Most AM proteins show a low or medium degree of conservation with respect to the three evolutionary markers used (i.e., a *v *similar to *fibrinopeptide*). However, there is a small group of proteins (< 10%) characterized by a medium to high degree of conservation with a *v *value ranging between that of *histone H4 *and SOD2. These conserved proteins are involved in cell cycle regulation (e.g., AKT3, members of the *cullin *family, GSK3B, SRC), signal transduction (e.g., HRAS, MAPK, PDCD6, YWHAE, YWHAG), protein metabolism and post-translational modifications (e.g., BECN1, MASK, RPL5, STK25) (Figure 1, Panel C).

#### AM proteomics

**Figure 2:**
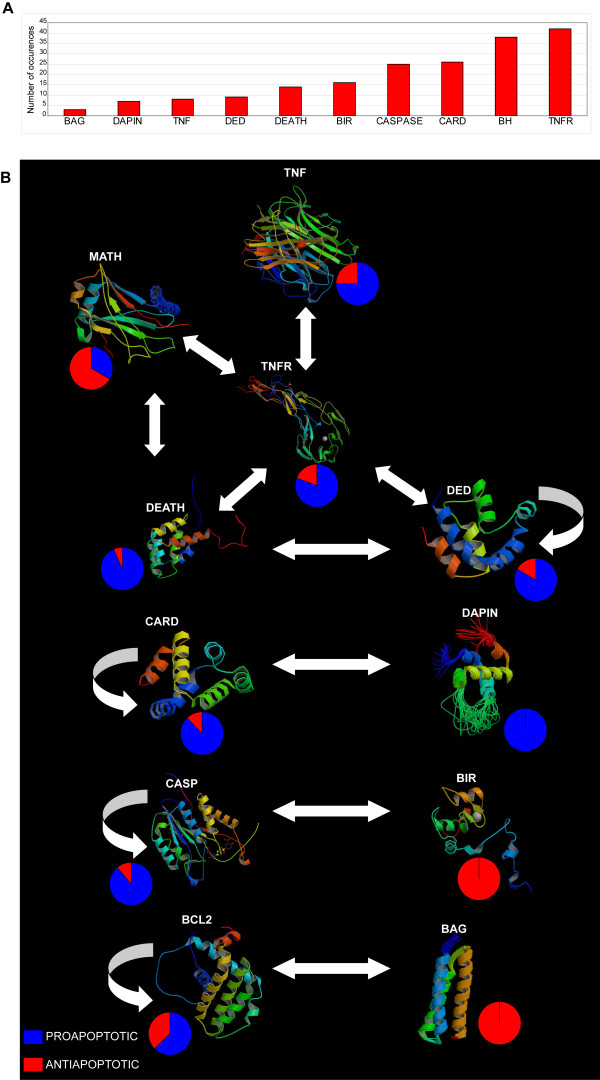
**Apoptotic structural modules**. Panel A. Most frequent motifs and domains in AM. Panel B. Map of interactions among the most frequent apoptotic structural modules and their propensity to perform a negative or positive control of cell death.

The molecular weight (MW) of AM proteins ranges between 4,430 daltons (isoform 6 of E2F6) and 527,622 daltons (BIRC6). About 20% of these proteins have a MW of approximately 20 kD (Additional file 7). Analysis of the post-translational modifications of AM proteins from the database HPRD showed that the most frequent are phosphorylation (35%) and proteolytic cleavage (15%). For 25% of these proteins there is no PTM information (Additional file 7). About 16% of AM proteins are metallo-proteins containing zinc (9.5%), calcium (3.2%), magnesium (2.9%), iron ions (0.9%) (Additional file 7). By scanning their primary structure, we identified 113 different motifs or domains with a total of 471 different structural modules: as expected, many AM proteins comprise more than a single module (Additional file 8). Direct structural characterization demonstrated that about 6.5% of AM proteins are NUPs [56]. With the exclusion of structural modules widespread within the human proteome (as the Protein Kinase domain and ANK Repeats), the most frequent structural motifs (i.e., BH, CARD, CASP, TNFR) are linked to AM *core *functions such as reception, transduction and execution of death signals (Figure 2, Panel A). By overlapping these data to the interactome, it is possible to identify a hypothetical pathway of interactions among protein domains, coupling it to their propensity to perform anti- or pro-apoptotic functions (Figure 2, Panel B). This analysis confirms previous literature data, suggesting that the death domains CARD, DAPIN, DEATH, DED can mediate and modulate both the reception as the transduction of apoptotic signals originating from the plasma membrane (TNFR domains), and of those incoming from mitochondria (BCL2 domains in all their variants resulting from recombination of BH motifs), up to the final effectors (CASP domains) (Figure 2, Panel B) [68]. The death domains may reciprocally interact as homomers or heteromers (CARD and DED), as they do with other protein domains that control their signal transduction activity (i.e., MATH) (Figure 2, Panel B). Core AM nodes are negatively regulated by: (1) the BAG domain, which negatively modulates the BCL2 domains of pro-apoptotic proteins; (2) the BIR domain, which represses the CASP domains [68]. This model is based on high-throughput interactome data (see Methods) and is consistent with other motif interaction maps [69-72], even though some of its specific details are different respect to low-throughput data [6,21,73]. Typically, the comparison between HT analysis and tests performed gene by gene (or protein by protein) results in a mismatched set of data [74,75]. This is especially true for interactome data. The very large spectrum of the analysis is generally coupled to low-confidence results. However, as demonstrated by a large amount of literature [22,76], the worth of HT data remains valid because they allow us to observe the behaviour of a whole system (a cell or a molecular machinery).

#### AM interactomics and molecular networks

**Figure 3:**
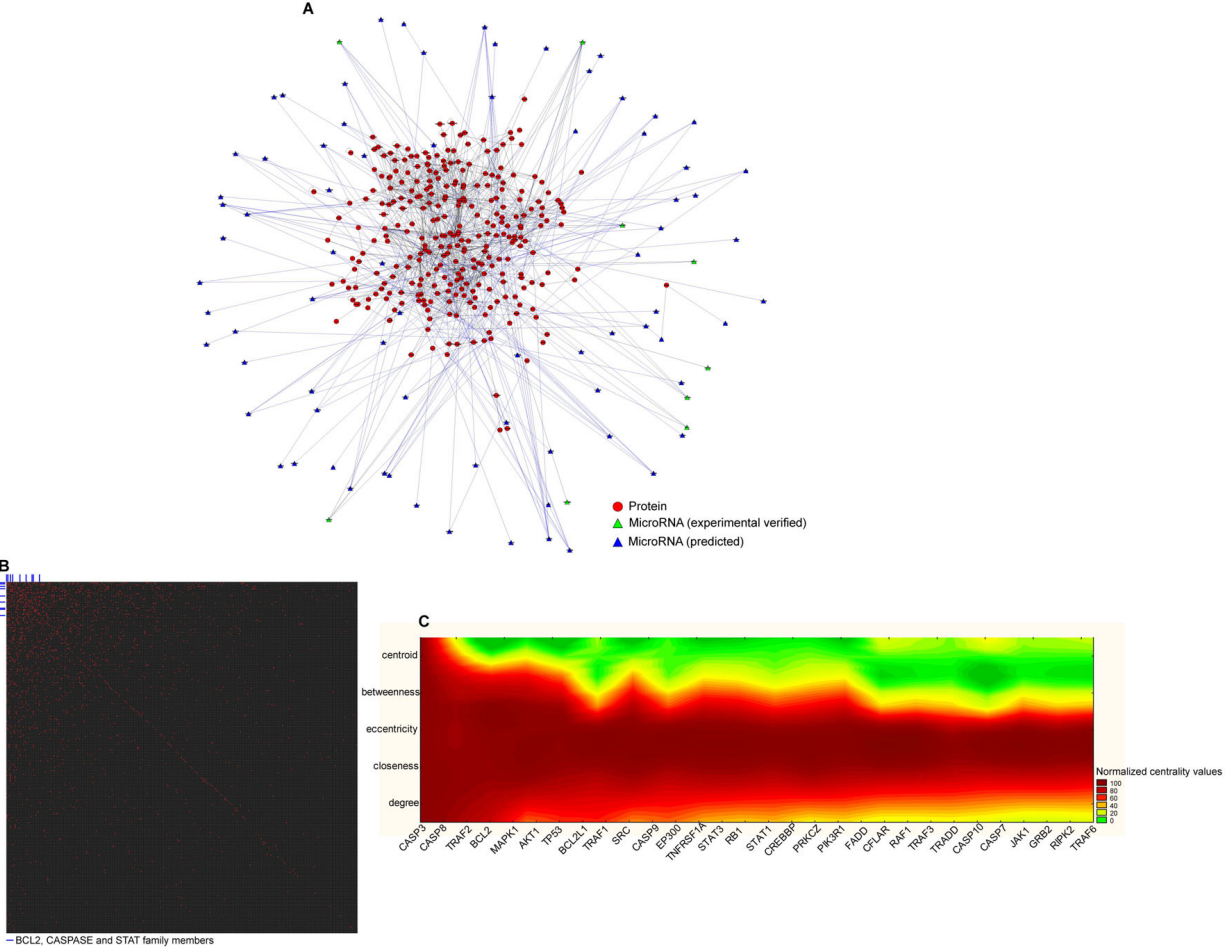
**Interactomics of AM**. Panel A. AM Network: red circles represent the proteins; green triangles represent experimentally verified microRNAs; blue triangles represent predicted microRNAs; blue lines represent the interactions (protein-protein, protein-DNA, microRNA-mRNA interactions). Panel B. Matrix of interactions of AM. Each axis represents all AM proteins and the red dots indicate the presence of an interaction of a given pair of proteins. The blue lines point out the BCL2, CASPASE and STAT family members. Panel C. Heat map of centrality values of AM proteins: the X-axis represents the AM proteins with the highest centrality, while the Y-axis represents different centrality parameters (betweenness, centroid, closeness, degree, eccentricity). The colours indicate the centrality levels, according to the colour bar shown on the right of the matrix.

**Figure 4:**
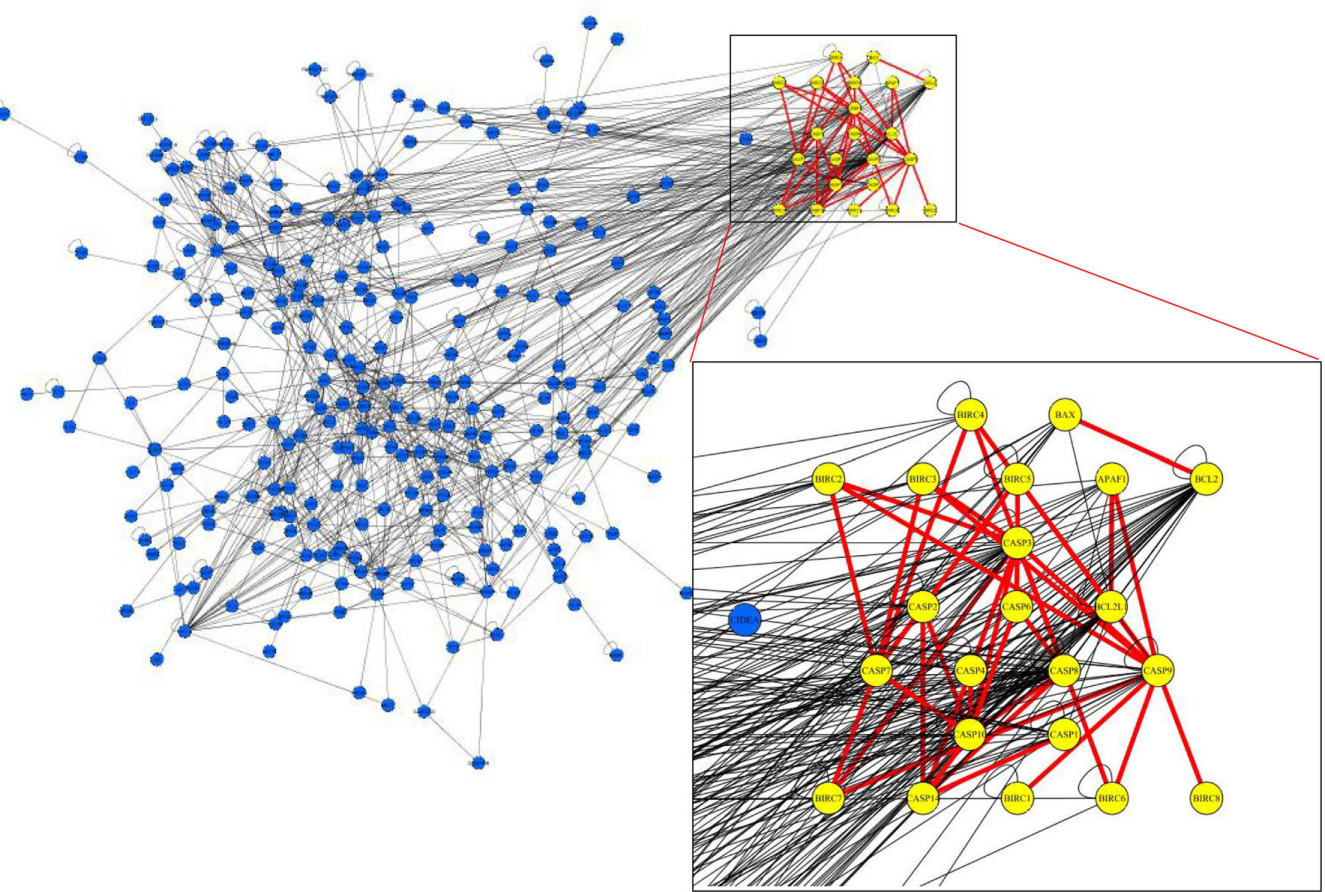
**The most evolutionarily conserved interactions in AM**.

According to BIND and HPRD data, protein-protein and protein-nucleic acids interactions are known for 86% of AM genes for which we found 1012 interactions (Figure 3, Panel A). The AM network is structurally and functionally based on the interactions among members of the BCL2, Caspase, and STAT families, which comprise about 42% of all links (Figure 3, Panel B). Its hubs, interacting with more than 10% of their neighbours, are CASP3 (13.5%), CASP8 (12.4%), TRAF2 (12%), and BCL2 (11%). By considering different centrality measures (betweenness, centroid, closeness, degree, eccentricity), the most central AM nodes are AKT1, CASP3, CASP8, MAPK1 (Figure 3, Panel C). These nodes, as many others with high centrality such as BCL2 or TRAF2, represent lethal embryonic perinatal or lethal postnatal genes in the mouse (MGI phenome data). Inside the AM network we identified two clusters of highly interconnected nodes: (a) the BCL2 family interaction cluster (BAX, BAK1, BCL2, BCL2L1, and BCL2L10); (b) the STAT family interaction cluster (CSF2RB, EPOR, NMI, STAT1, STAT3, STAT5A, and STAT5B). This could be potentially important for understanding the functional relationships among these gene in physiology and pathology. By comparing the human AM network with that of *M. Musculus*, *C. elegans*, *D. Melanogaster*, we found that the interactions between BCL2/APAF1, BCL2/BAX, BIRCs/CASPs, CASP9/APAF1, and CASPs/CASPs were conserved in these organisms (Figure 4). Interestingly, we failed to find any significant correlation between network centrality and specific phenotypic features.

### Pathology

#### AM oncogenomics

**Figure 5:**
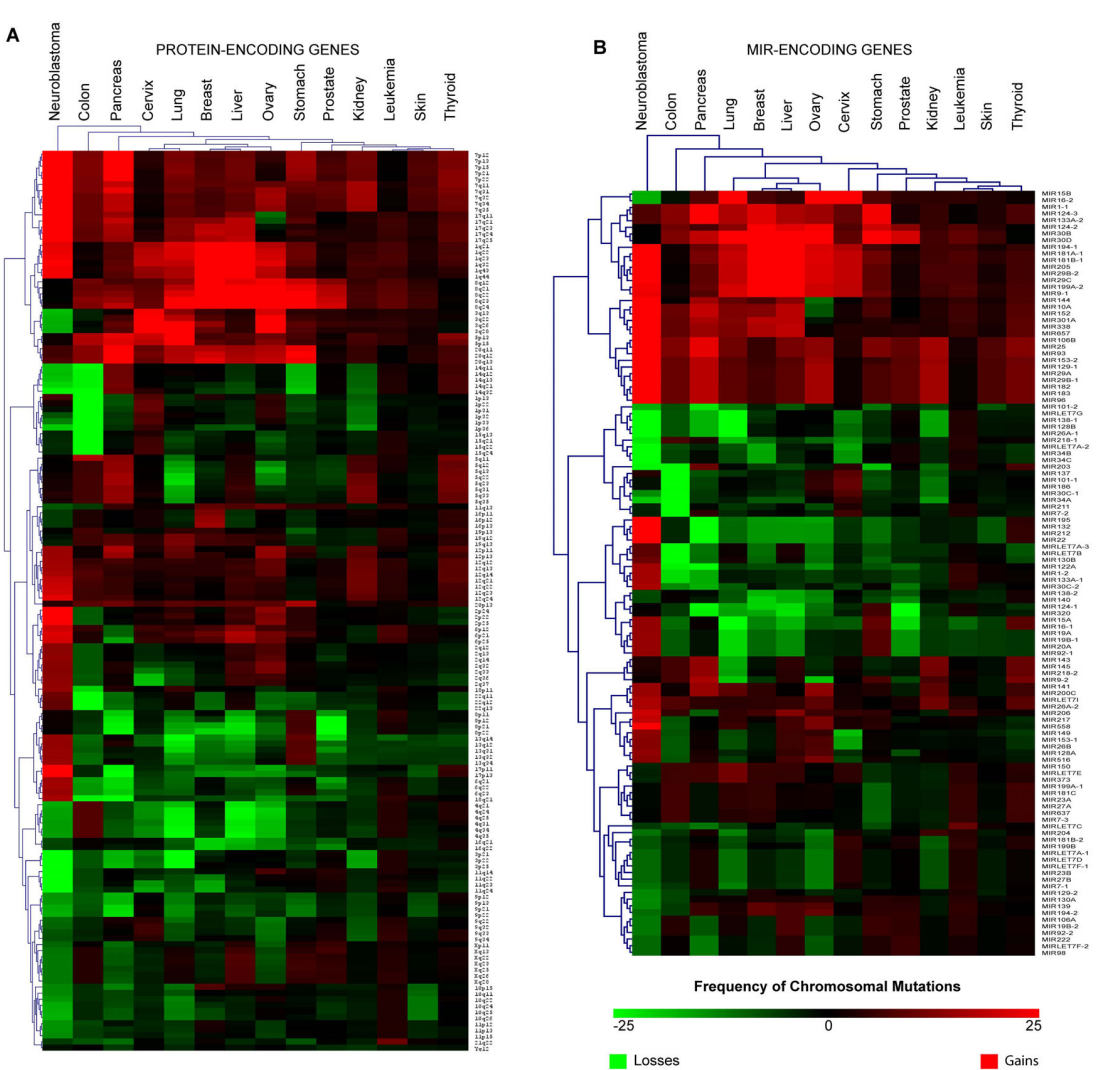
**Frequency of chromosomal mutations involving AM loci**. Panel A. Heat Map showing the frequencies of chromosomal mutations related to AM protein-encoding-loci in the fourteen cancer models (see Methods). Panel B. Heat Map showing the frequencies of chromosomal mutations related to MIR-encoding loci for AM in fourteen cancer models. Red indicates genome amplification, while green points to genome deletions.

**Figure 6:**
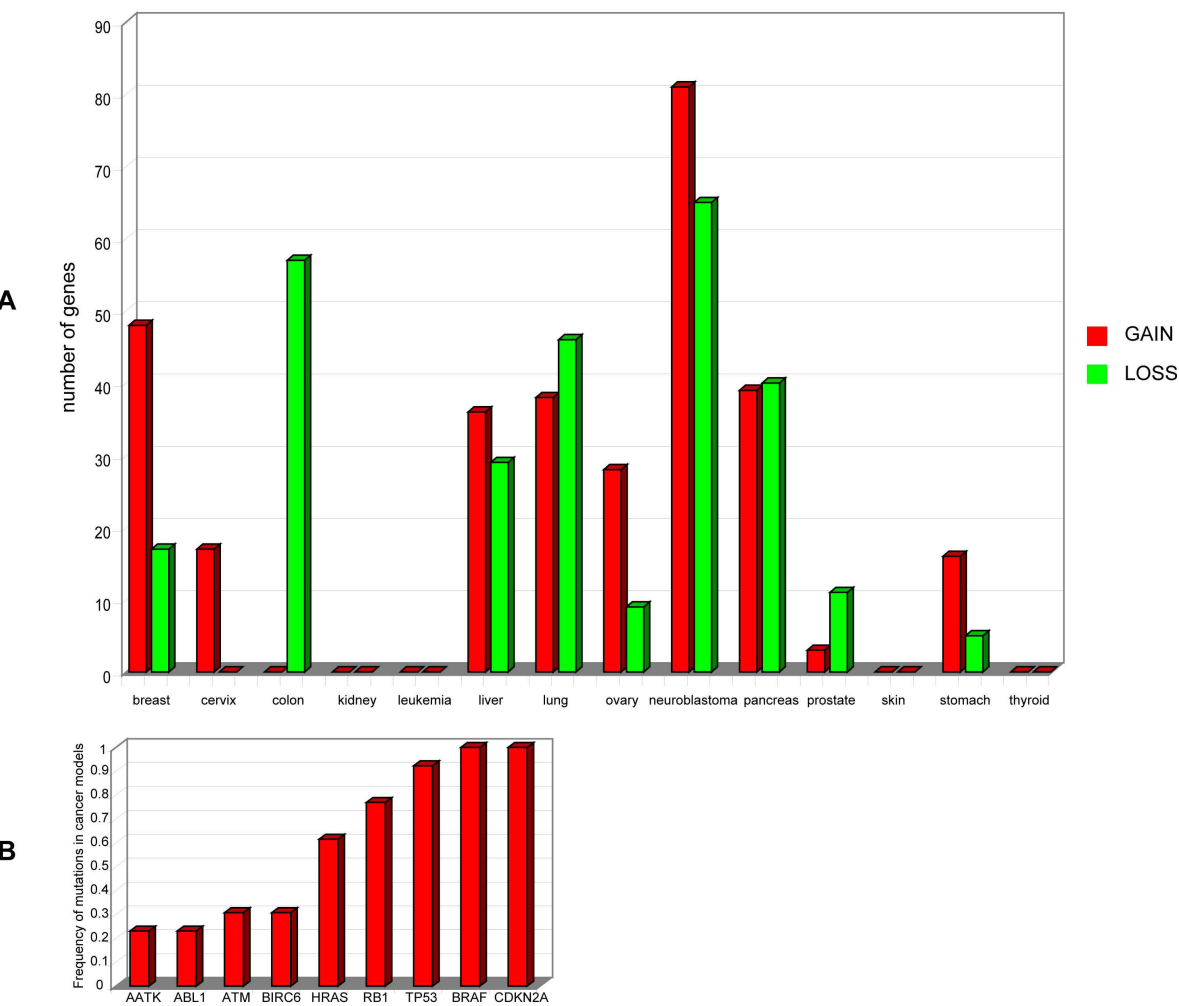
**Mutations of AM genes**. Panel A. Number of AM genes involved in cancer related GI. Red and green bars show the genome amplifications and deletions, respectively. Panel B. Frequency of AM genes point mutations in cancer.

By overlapping the AM genome map with the chromosome aberration map in tumours, we found that AM protein-encoding genes at 8q23 and 8q24 are potentially involved in gain-type mutations (duplication or amplification) with a frequency higher than 20% in about 50% of cancers. This also occurs with AM genes at 1q23 and 1q32 (about 40% of cancers). On the contrary, AM genes at 8p21 and 8p22 are located in regions that are loss-prone in 35% of cancers (Figure 5, Panel A; Additional file 9). The most represented cancers in this amplification/deletion AM map are neuroblastoma (146 AM protein encoding genes), lung (84 AM protein encoding genes), pancreas (79 AM protein encoding genes) (Figure 6, Panel A). MIRs regulating the expression of AM genes (most frequently endowed with anti-apoptotic functions) also are localized in regions frequently altered in cancer: MIR9-1, MIR29B2, MIR29C, MIR181B1, MIR199A2, MIR205, MIR213 map to chromosome 1 long arm, a gain-prone region in 6 out of 14 of cancer models; the same is true for MIR30B and MIR30D, both localized at 8q24.22, a gain-region in 7/14 of cancer models (Figure 5, Panel B). On the other hand, MIR124A1 and MIR320 are localized in loss-prone regions in 5/14 of the models (Figure 5, Panel B). Screening of databases for somatic mutation in cancer demonstrated that the most frequently mutated AM protein-encoding genes are the protooncogene BRAF (mutated in 100% of cancer models) and the tumour suppressors CDKN2A and TP53. The latter two are altered in 100% and 90% of cancer models analyzed, respectively (Table 3; Figure 6, Panel B). BRAF is frequently subject to point mutations in skin and thyroid cancers (41% and 36%, respectively), but at much lower frequencies in the other tumour types (1–5%). CDKN2A is mutated in about 10–15% of samples, with the highest incidence in pancreas tumours. Point mutations were detected at the TP53 locus with an incidence of about 50% in each tumour model, with a maximum of 83% in thyroid neoplasms (Figure 6, Panel B). Intriguingly, BRAF, CDKN2A, TP53, RB1 are frequently co-mutated in the same cancer types, strongly suggesting that this co-occurrence could be important for activation or progression of the neoplastic process (Table 3).

**Table 3:** AM genes mutated in specific cancers

**Breast**	ATM – BIRC6 – BRAF – BRCA2 – CDKN2A – CHUK – DAPK1 – HRAS – HUWE1 – NFKB1 – NFKBIA – PTPN13 – RB1 – STAT1 – TP53
**Colon**	BRAF – BRCA2 – CDKN2A – RB1 – SRC – TP53

**Kidney**	ATM – AVEN – BRAF – CASP3 – CASP9 – CDKN2A – CUL3 – HRAS – MAPK8 – RB1 – TP53

**Leukemia**	ABL1 – BRAF – CDKN2A – HRAS – RB1 – TP53

**Liver**	BRAF – BRCA2 – CDKN2A – RB1 – TP53

**Lung**	AATK – ABL1 – APAF1 – ATM – BCL2L13 – BIRC4 – BIRC6 – BIRC8 – BNIP3 – BRAF – BRCA2 – CARD6 – CASP14 – CASP4 – CASP5 – CDKN2A – CHUK – CREBBP – CUL4B – CYCS – DAPK3 – FASTK – HRAS – IRAK3 – MAP3K14 – MYBL2 – NTRK1 – P53AIP1 – PAX7 – PDCD5 – RAF1 – RB1 – RET – SGK – SGK3 – STK17B – STK3 – TGFBR2 – TNFRSF9 – TP53

**Neuroblastoma**	BRAF – CDKN2A – TP53

**Ovary**	AATK – BRAF – BRCA2 – CDKN2A – DAPK3 – MAP3K14 – NTRK1 – RAF1 – RB1 – RET – STK3 – TP53

**Pancreas**	BIRC6 – BRAF – CDKN2A – FAF1 – TP53

**Prostate**	BRAF – CDKN2A – HRAS – RB1 – TP53

**Skin**	ABL1 – BAX – BCL2L13 – BCL2L14 – BIRC3 – BIRC6 – BNIP3 – BRAF – BRCA2 – CARD14 – CARD6 – CASP10 – CASP4 – CASP8 – CD40 – CDC2L1 – CDKN2A – CORO1C – CREBBP – CUL4A – DAXX – E2F6 – ENC1 – HIPK2 – HRAS – IL4R – MAPK7 – MYCN – PDCD4 – PDCD6IP – RB1 – RIPK1 – TNF – TNFRSF1A – TNFRSF8 – TNFRSF9 – TNFSF10 – TNFSF13 – TP53 – TP73L – TRAF2

**Stomach**	AATK – ATM – BRAF – CDKN2A – FASTK – HRAS – JAK1 – NTRK1 – RB1 – SGK – TGFBR2 – TP53

**Thyroid**	BRAF – CDKN2A – HRAS – RET

#### AM epigenomics

According to PubMeth, DAPK1, PYCARD, RB1, and TP73 are methylated in at least half of our fourteen cancer models (Table 4). According to our transcriptome data, DAPK1 (reported to be a tumor suppressor candidate) is down expressed in breast, colon cancer, leukemia, neuroblastoma and is methylated in all models analyzed. However, unlike other hypermethylated genes, DAPK1 is localized in a genomic region that is never loss-prone in cancer. This could suggest that its down regulation, possibly relevant in some cancer phenotypes, may be obtained through aberrant methylation rather than genome mutations, possibly because the deletion of its chromosomal region could impair the vitality of the cell.

**Table 4:** Methylations of AM genes associated to cancer

**GENE**	**Breast**	**Colon**	**Kidney**	**Leukaemia**	**Liver**	**Lung**	**NB**	**Ovary**	**Pancreas**	**Prostate**	**Skin**	**Stomach**	**Thyroid**
**ABL1**				47									

**APAF1**			98	35									

**ATM**		10											

**BCL2**	18	22								52			

**BCL2L10**												80	

**BDNF**												80	

**BIRC4**		75										75	

**BNIP3**		66		16						80		49	

**BRCA2**								5					

**CASP8**					100		37						

**CD44**							20			33			

**CDKN1A**				41						8			

**CFLAR**							5						

**DAPK1**	55	47	32	16	7	34	16	67	14	16	19	51	24

**DIABLO**				25									

**DUSP6**									42				

**FAS**		40								13			

**FOXE1**									70				

**GADD45A**	60												

**GADD45G**		20		33	20	40						11	

**GPR37**				72									

**GSK3B**		2											

**HRAS**	100												

**HRK**		14										17	

**IGFBP3**	18		38			70		43			32	71	

**PYCARD**	30	25		8	36	46	31	19		52	63	5	

**RB1**	17			14	25	19	21			8	5	42	

**TGFBR2**	30									100			

**TNFRSF10A**							48						

**TNFRSF10C**	70			26		23	25	31		65			

**TNFRSF10D**	74					28	31			25			

**TP53**	12				13								

**TP73**	50	61		20	22		29		14			29	

Numeric values correspond to the mean of methylation degree (% of sample where methylation was detected in primary samples, according to Pubmeth).

#### AM transcriptomics: protein-encoding genes

**Figure 7:**
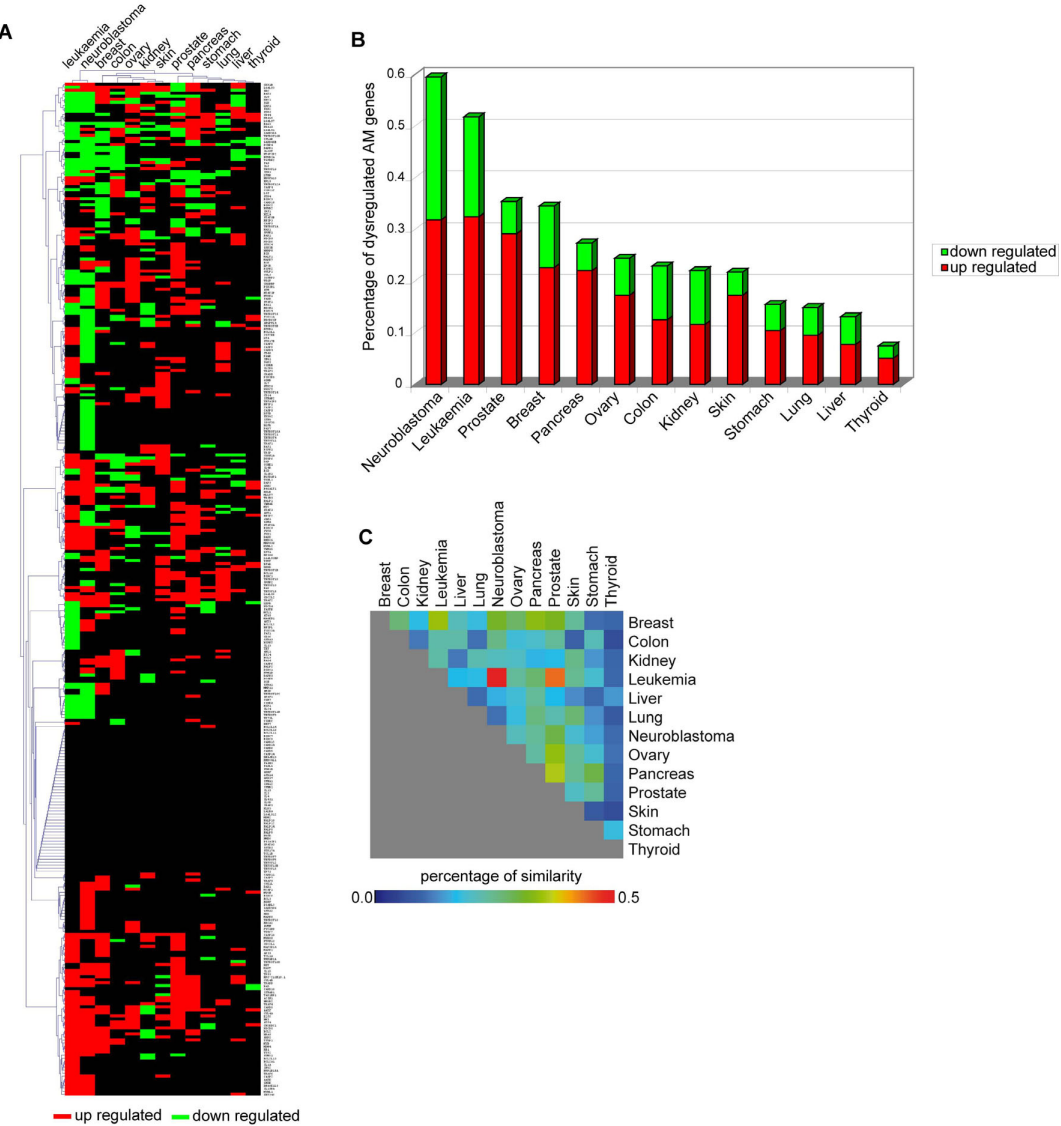
**AM trascriptome in cancer**. Panel A. Expression matrix of protein-encoding AM genes in cancer. Up regulated genes are depicted in red, down regulated genes in green. Each row represents the colour coded expression of a specific gene in the indicated cancer models; the columns represent the colour coded AM profiles of each cancer model. The dendrogram above the matrix represents the clusters of the tumours. Panel B. Percentage of dysregulated AM genes in cancer. Panel C. Matrix of identity profile of AM dysregulation in cancer, based on number of the same dysregulated genes for each couple of models normalized for their average number of altered genes.

**Figure 8:**
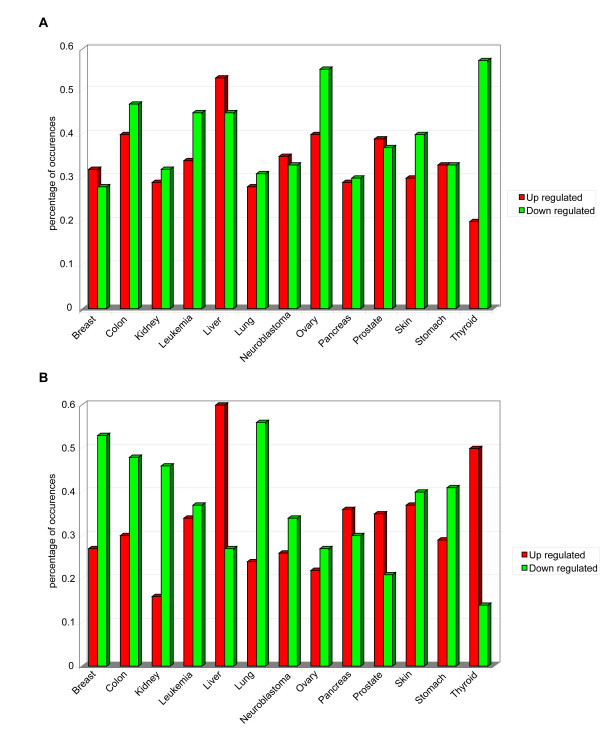
**Transcriptome alteration of regulators of apoptosis**. Panel A. Positive regulators of AM. Panel B. Negative regulators of AM.

By performing the normalization and discretization of the expression of AM protein-encoding genes in breast, colon, kidney, leukaemia, liver, lung, neuroblastoma, ovary, pancreas, prostate, skin, stomach, and thyroid, we identified those that are transcriptionally deregulated in these tumours (Figure 7, Panel A; Additional file 10). Expression of these AM genes is frequently altered in neuroblastoma and leukaemia (about 60% and 52%, respectively) (Figure 7, Panel B). Our analysis showed that AATF, LGALS3, and SRC are up regulated in 8/13 of cancer models, and CDC2L2, E2F6, LGALS9, PDCD8, RELB, TRADD, and TRAF2 in 7/13 (Figure 7, Panel A; Additional file 10). On the other hand, the most frequently down regulated AM genes are TGFBR2 (7/13 of cancer models), BAG3, CLU, LGALS1, LTBR, SGK (in 6/13) (Figure 7, Panel A; Additional file 10). Notably, comparison of AM transcriptome patterns suggests that each cancer model has its own specific AM transcriptome profile, even if some (e.g., leukemia and neuroblastoma) showed similar patterns (Figure 7, Panel C). Not surprisingly, the positive regulators of apoptosis tend to be down regulated in all cancer models (in particular in cancers of the ovary and thyroid); also the negative regulators of apoptosis are generally down regulated in most models, except for pancreas, prostate, thyroid cancers in which they are up regulated instead. These data confirm the complexity of the cancer genome alterations and further emphasize the need for a System Biology approach to its pathogenesis and therapy (Figure 8, Panels A, B).

#### AM transcriptomics: MIR-encoding genes

**Figure 9:**
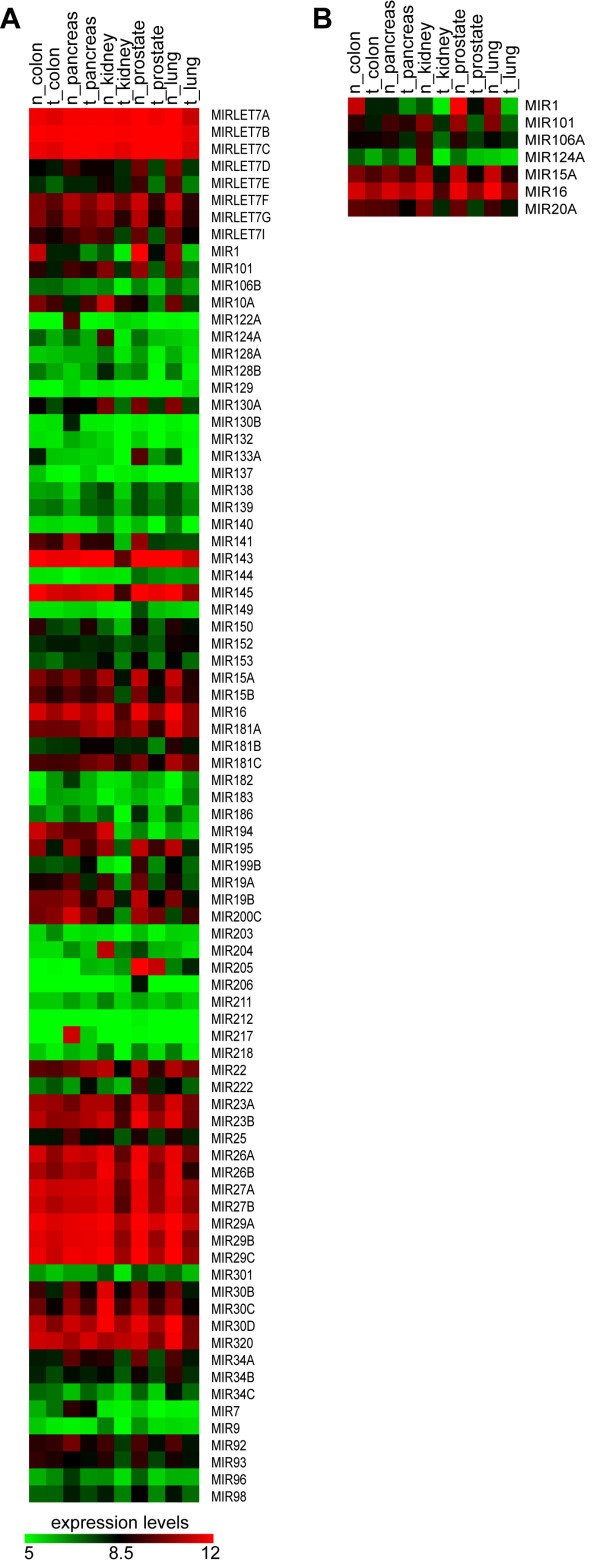
**Trascriptome of AM MIRs in cancer**. Panel A. Expression matrix of MIR-encoding genes computationally predicted as targeting AM. Panel B: Expression matrix of MIR-encoding genes experimentally validated as targeting AM. The colours indicate the expression levels of MIRs according to the bar shown above the matrixes. n = normal; t = tumoral.

By searching the database VITA, we determined the expression profile of MIRs predicted to target AM proteins in colon, kidney, lung, pancreas, prostate cancers (Figure 9, Panels A, B). Our analysis demonstrated that different AM proteins, that are up regulated in different cancers, are computationally predicted targets of MIRs, that are down regulated in the same tumours (Table 5). In particular, CUL3, over expressed in kidney and prostate cancers, is a target of several dysregulated MIRs: MIR22, MIR23A, MIR23B, MIR218-1, MIR218-2, and MIR301, which are down regulated in kidney cancers, and of MIR22, MIR23A, MIR181A, and MIR181C, which are down regulated in prostate cancers (Table 5). These data supply a list of new candidate MIRs possibly involved in cancer (Table 5). Our proposal is strengthened by the presence, within this list, of MIRs that were already experimentally demonstrated to target AM protein encoding genes (Additional file 3). In fact, we found that in lung cancer over expression of BCL2 could be explained by the under expression of MIR15A, MIR16-1, and MIR16-2, while in kidney tumours over expression of DFFB, HTATIP and RELA could be related to the under expression of MIR124A-1, MIR124A-2, and MIR124A-3 (Table 5). Similar to the AATK gene that contains it, also MIR338 is up regulated in neuroblastoma (M Ragusa et al., 2009, submitted).

**Table 5:** Transcriptome and proteome alterations of AM targets of MIRs

**GENE**	**mRNA**	**PROTEIN**	**MIRs**
**BCL2**	Up regulated in LUNG	Up regulated in LUNG	MIR15A, MIR16-1, MIR16-2, MIR34A down regulated in LUNG

**CUL3**	Up regulated in KIDNEY, PROSTATE	Up regulated in KIDNEY, PROSTATE	MIR22, MIR23A, MIR23B, MIR218-1, MIR218-2 MIR301 down regulated in KIDNEY; MIR22, MIR23A, MIR181A, MIR181C down regulated in PROSTATE

**DFFB**	Normal in KIDNEY	Up regulated in KIDNEY	MIR124A-1, MIR124A-2, MIR124A-3 down regulated in KIDNEY

**HTATIP**	Normal in KIDNEY	Up regulated in KIDNEY	MIR124A-1, MIR124A-2, MIR124A-3 down regulated in KIDNEY

**IL1A**	Up regulated in LUNG; normal in PROSTATE	Up regulated in LUNG; normal in PROSTATE	MIR30B down regulated in LUNG and PROSTATE; MIR30D down regulated in LUNG

**IRF1**	Normal in LUNG, KIDNEY, PANCREAS	Up regulated in LUNG, KIDNEY, PANCREAS	MIR130A, MIR130B down regulated in LUNG, KIDNEY, PANCREAS; MIR23A down regulated in LUNG, KIDNEY

**NGFR**	Normal in KIDNEY	Up regulated in KIDNEY	MIR7-1, MIR7-2, MIR7-3, MIR128A, MIR128B down regulated in KIDNEY

**PDCD4**	Down regulated in LUNG	Up regulated in LUNG	MIR15B, MIR16, MIR145, MIR195 down regulated in LUNG

**RB1**	Normal in KIDNEY	Up regulated in KIDNEY	MIR98, MIRLET7G down regulated in KIDNEY

**RELA**	Normal in KIDNEY	Up regulated in KIDNEY	MIR124A-1, MIR124A-2, MIR124A-3 down regulated in KIDNEY

**STAT3**	Normal in KIDNEY	Up regulated in KIDNEY	MIR106 down regulated in KIDNEY

**TP73L**	Down regulated in LUNG; normal in PANCREAS	Up regulated in LUNG, PANCREAS	MIR92 down regulated in LUNG, PANCREAS

#### AM genomics vs transcriptomics

**Figure 10:**
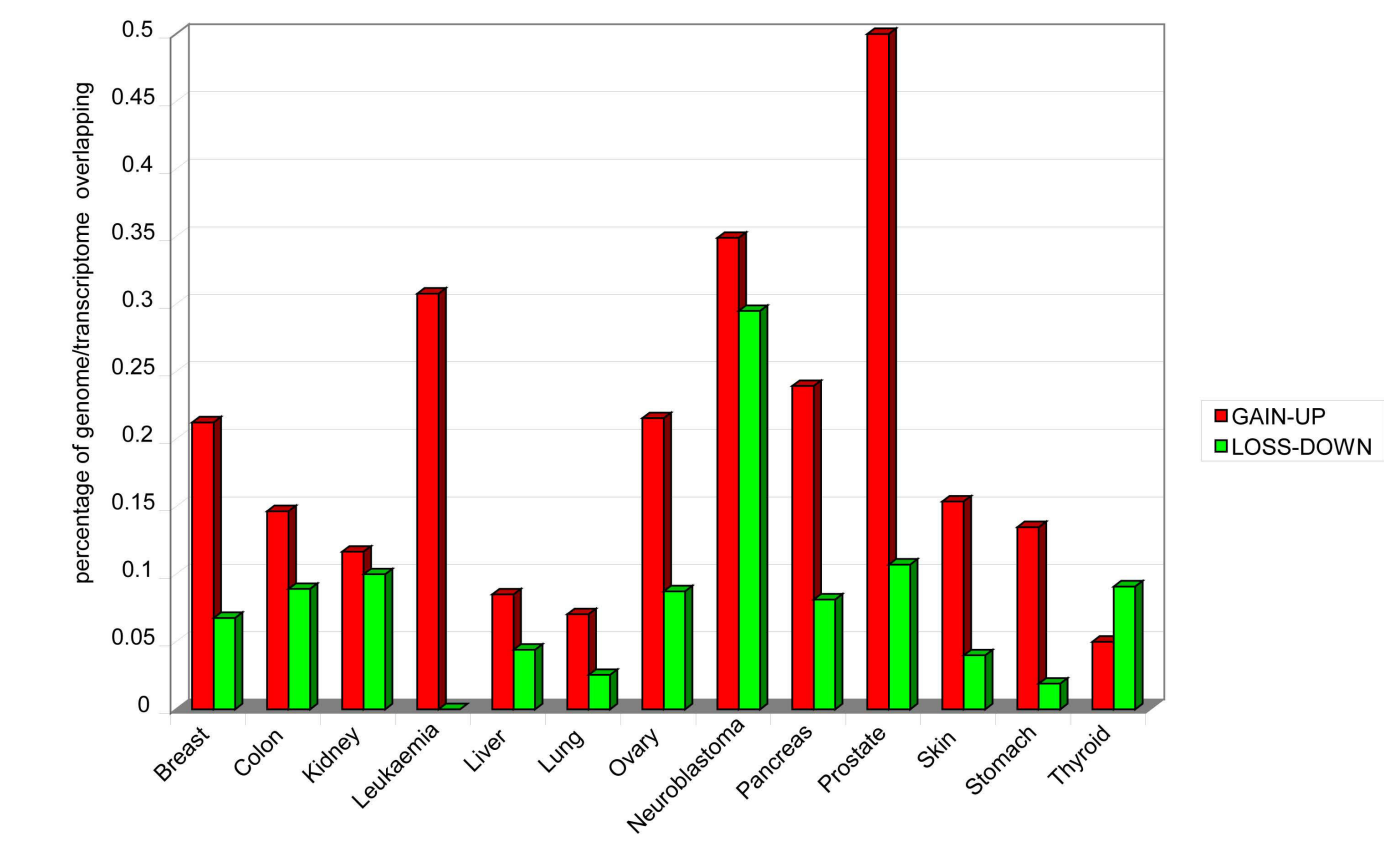
**Overlapping between AM genome alterations and transcriptome dysregulation in cancer**. Percentage of AM genome/transcriptome alterations in cancer models.

Overlapping the chromosomal mutation map to AM expression profiles in each cancer model showed that 28% of AM genes with transcriptional alterations were located in genomic regions potentially mutated in neoplasia. The highest rate of overlapping was found in prostate cancers (50% in regions of gain, 11% in regions of loss) and neuroblastoma (35% in regions of gain, 29% in regions of loss), whilst the lowest was in lung (7% in regions of gain, 2.5% in regions of loss) and liver cancers (8.5% in regions of gain, 4.4% in regions of loss) (Figure 10; Additional file 11). We overlapped the MIR transcriptome to CGH-array data in colon, kidney, lung, pancreas, and prostate tumours. Prostate cancer is the one with the highest number of altered genomic regions and concomitant aberrant expression of MIR genes (50% of regions with gain- or loss-mutations also show up- or down regulation of their MIRs). In many cases we found that down regulation of a MIR could correspond to the heterozygous deletion of the encoding gene (Table 6).

**Table 6:** MIR genes localized in rearranged genome regions and transcriptionally altered

**Genome/Transcriptome**	**COLON**	**KIDNEY**	**LUNG**	**PANCREAS**	**PROSTATE**
**GAIN/UP**				MIR10A	

**GAIN/DOWN**			MIR133A2*, MIR1-1*	MIR1-1*	

**LOSS/DOWN**	MIR1-2*, MIR133A1*, MIR30C-1	MIRLET7G, MIR128B	MIRLET7F1, MIRLET7G, MIRLET7D, MIR101-2, MIR10A, MIR128B, MIR143, MIR152, MIR15A, MIR15B, MIR16-1, MIR195, MIR199B, MIR19A, MIR19B1, MIR218-1, MIR218-2, MIR23B, MIR26A-1, MIR29C, MIR30B, MIR30D, MIR320, MIR34A, MIR34B	MIR130B, MIR141, MIR182, MIR19A, MIR19B1, MIR19B2, MIR200C, MIR222, MIR92A1, MIR92A2	MIR15A, MIR16-1, MIR19A, MIR19B1

The symbol * indicates those mature MIRs that could be encoded by different pre-MIR genes, localized in different genomic positions.

#### AM proteomics

**Figure 11:**
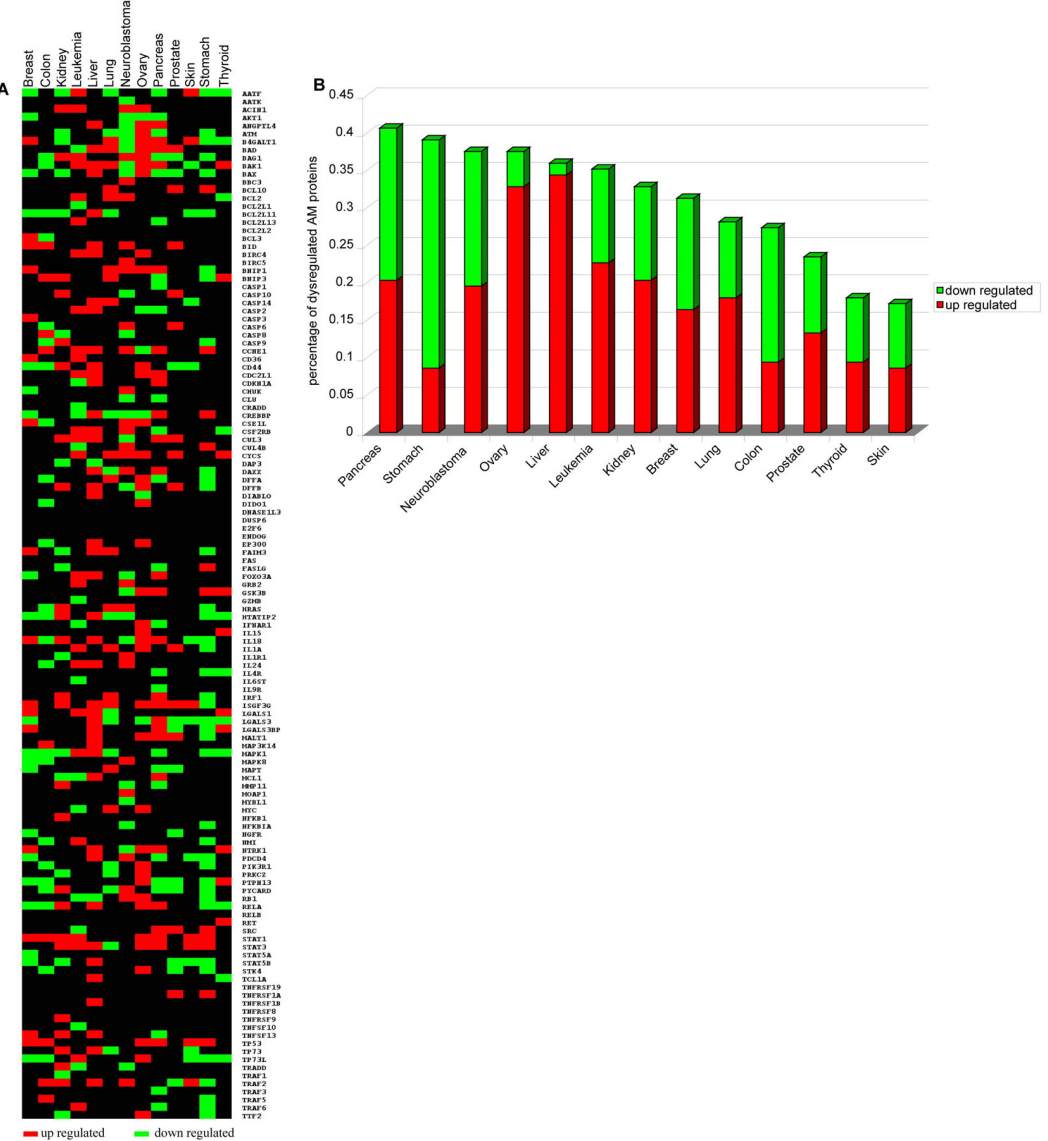
**AM proteomics in cancer**. Panel A: Expression matrix of AM proteins in cancer. Up regulated proteins are depicted in red, down regulated proteins in green. Panel B: Percentage of dysregulated AM proteins in cancer.

**Figure 12:**
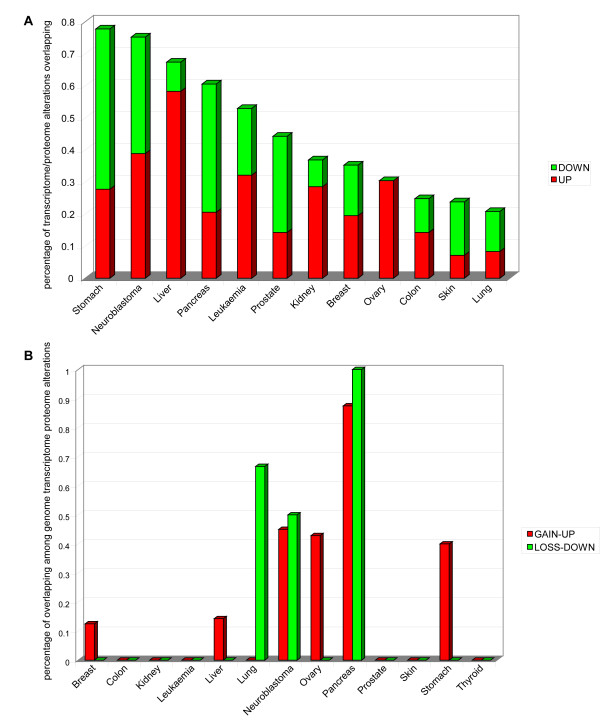
**Superimposition of genome/transcriptome/proteome alterations of AM genes**. Panel A: Overlapping between AM transcriptome and proteome dysregulations in cancer. Panel B: Overlapping between AM genome, transcriptome and proteome alterations in cancer.

**Figure 13:**
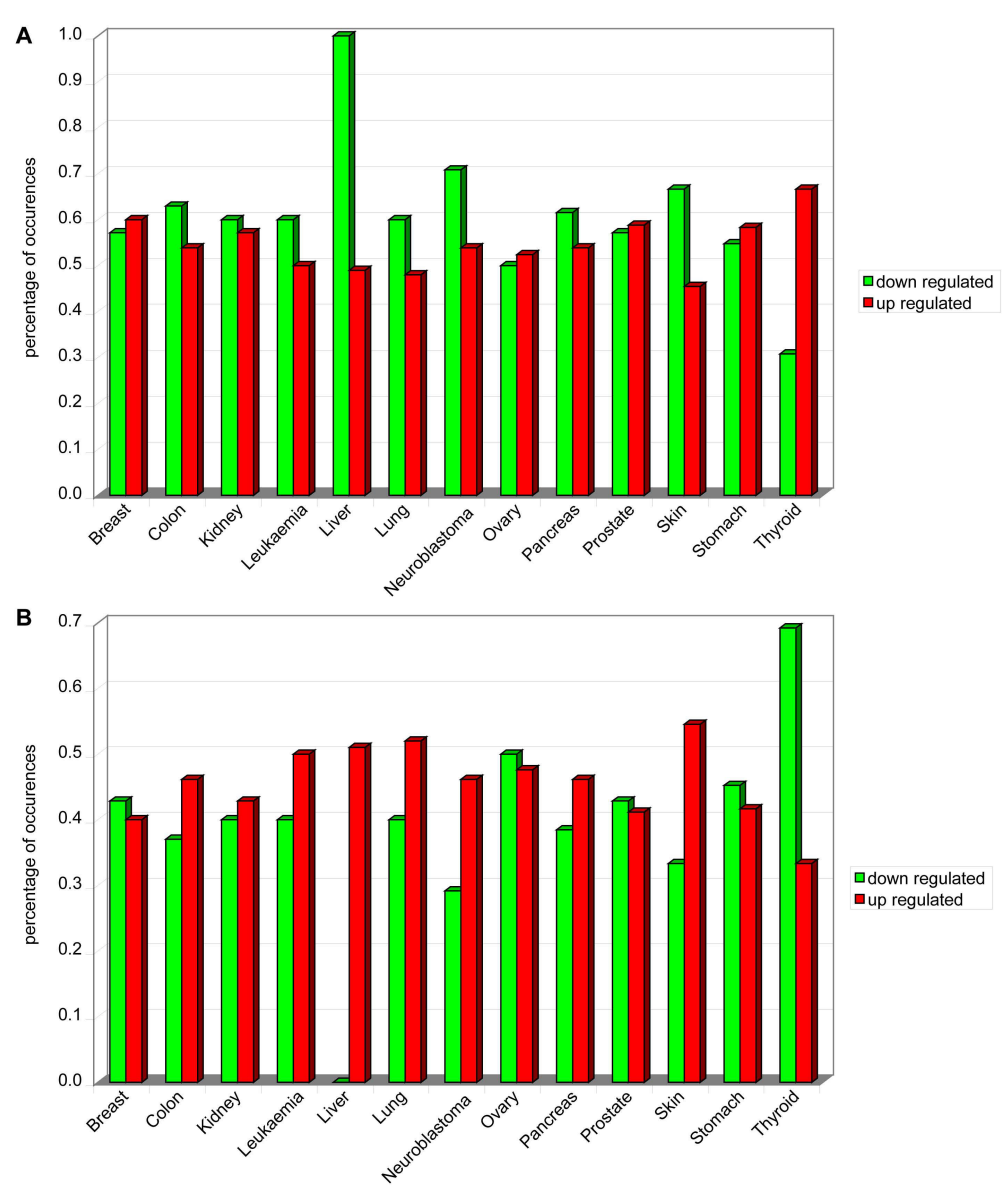
**Proteome alteration of regulators of apoptosis**. Panel A: Positive regulators of AM. Panel B: Negative regulators of AM.

By performing the normalization and discretization of AM protein expression in cancers of the breast, colon, kidney, leukaemia, liver, lung, neuroblastoma, ovary, pancreas, prostate, skin, stomach, thyroid, we identified a set of proteins that are dysregulated in these tumours (Figure 11, Panels A, B). EP300, ISGF3G, STAT1, and STAT3 are up regulated in 8/13 of cancer models, while ATM, BAX, BCL2L11, HTATIP2, LGALS3, MAPK1, and TP73L are down regulated in half of cancer models (Figure 11, Panel A). By overlapping protein expression data to transcriptome data, we identified the AM genes with transcriptome alterations mirroring their proteome dysregulation (Table 7). Because of this degree of consistency, these genes may be assumed to represent relevant cancer candidates in different tumour models. We found that this correlation was more evident in leukemia (17.4% of up regulated, and 7.6% of down regulated), liver cancer (27% of up regulated, and 5.5% of down regulated), and neuroblastoma (18.7% of up regulated, and 13% of down regulated) (Figure 12, Panel A). We also identified those genes that are potentially rearranged (both gain- or loss-type of mutation) and have altered transcriptome and proteome expression (Table 8). Cancers showing a noteworthy overlapping of genome mutations, transcriptome dysregulation and altered protein expression were pancreatic tumours and neuroblastoma for up- or down regulated AM proteins, and lung cancers for down regulated AM proteins (Figure 12, Panel B). Analysis of the function of dysregulated AM proteins showed that the positive regulators of apoptosis tend to be down regulated in all cancer models, whereas the negative regulators of apoptosis generally tend to be up regulated in most models (Figure 13, Panels A, B). The same analysis performed on AM genes with common transcriptome/proteome alterations showed a tendency to flattening of the anti/pro genes balance.

**Table 7:** AM genes with concordant transcriptome and proteome dysregulation in cancer

**BREAST**	BNIP1+, CSE1L+, EP300+, IL18+, LGALS3BP+, NTRK1+, STAT1+, TP53+, CD44-, RELA-, STAT5A-, STAT5B-
**COLON**	CDKN2A+, HTATIP2-, PTPN13-

**KIDNEY**	AATF+, ANGPTL4+, CASP10+, CASP9+, CCNE1+, CD44+, LGALS1+, LGALS3+, RIPK1+

**LEUKEMIA**	AATF+, ACIN1+, BAK1+, BCL2+, BCL2L13+, CASP2+, CCNE1+, CDC2L1+, CSF2RB+, CYCS+, FADD+, GRB2+, IL1A+, JAK1+, MAPK1+, NMI+, STAT1+, STAT3+, TRAF6+, CREBBP-, GZMB-, IL6ST-, MCL1-, TNFSF10-

**LIVER**	BAK1+, CD44+, EP300+, LGALS1+, LGALS3+, MAPK1+, TNFRSF1B+, DAP3-

**LUNG**	BAK1+, CD44+, EP300+, LGALS1+, LGALS3+, MAPK1+, TNFRSF1B+, DAP3-

**NEUROBLASTOMA**	ACIN1+, BBC3+, BCL2+, BID+, BIRC5+, CASP6+, CCNE1+, CHUK+, CSE1L+, CUL4B+, CYCS+, DAXX+, GRB2+, HRAS+, IL1R1+, MAPK8+, MOAP1+, PDCD4+, PYCARD+, RB1+, TRAF2+, AKT1-, ANGPTL4-, ATM-, CASP8-, CLU-, CREBBP-, CUL3-, DFFB-, HTATIP2-, MMP11-, NFKBIA-, NTRK1-

**OVARY**	ATM+, B4GALT1+, BAK1+, BAX+, NTRK1+, PIK3R1+, STAT1+

**PANCREAS**	BAD+, CD44+, LGALS3+, LGALS3BP+, RELA+, SRC+, STAT1+, STAT3+, CLU-, PDCD4-

**PROSTATE**	BAD+, BCL10+, CASP10+, CUL3+, CYCS+, FADD+, JAK1+, MALT1+, CD44-, LGALS3-, LGALS3BP-

**SKIN**	RIPK1+, TRAF2+, CD44-

**STOMACH**	BCL10+, CD44+, CDKN2A+, SRC+, STAT1+, STAT3+, PDCD4-

+ indicates up regulation, – indicates down regulation.

**Table 8:** AM genes mapping in frequently mutated genome regions with concordant transcriptome and proteome alterations in cancer

**BREAST**	**LIVER**	**LUNG**	**OVARY**	**PANCREAS**	**STOMACH**	**NEUROBLASTOMA**
NTRK1+	BAK1+	CD44-	BAK1+	LGALS3+	STAT3+	AKT1-
		AKT1-	NTRK1+	LGALS3BP+	SRC+	ANGPTL4-
			STAT1+	RELA+		ATM-
				SRC+		DFFB-
				STAT1+		HTATIP2-
				STAT3+		NFKBIA-
				BAD+		BCL2+
				PDCD4-		BID+
				CLU-		BIRC5+
						CSE1L+
						CYCS+
						DAXX+
						GRB2+
						IL1R1+
						RB1+

The sign + indicates the potential gain according to CGH data and up regulation in transcripts and proteins, while – indicates the potential loss according to CGH and down regulation of transcripts and proteins.

#### AM interactomics and molecular networks

**Figure 14:**
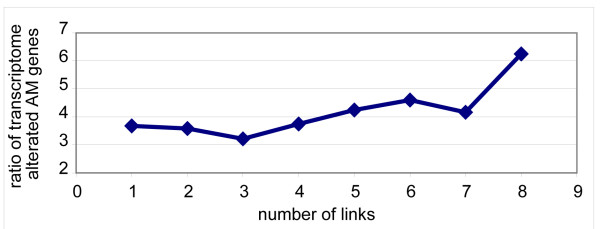
**Correlation between mutated links and transcriptome alterations**. The X-axis represents the distribution classes of AM network genes (without mutations), based on the number of the cancer mutated genes linked to a specific gene. The Y-axis represents the weighted average of the altered transcriptome AM genes for each class.

Analysis of the relationship between the link number of a gene to genome mutations and transcriptome alterations allowed us to discover that the genes with more links to mutated genes are more likely to be dysregulated in tumours (Figure 14). Our analysis demonstrates that the hubs of the AM network typically represent the nodes with the highest number of genome, transcriptome or proteome alterations in all cancer models analyzed, even though the oncogenic relevance of each hub seems to be tumour (or tumour group) – specific (Additional file 12). Moreover, we found that the average degree of the mutated nodes is significantly higher than the average degree of not mutated ones (p < 0.0001, Wilcoxon signed-rank test). Intriguingly, approximately 70% of NUPs are nodes with a higher degree of connectivity than the average AM proteins and some of them are hubs (i.e., AKT1, BCL2, BCL2L1, CDKN2A, and TP53): indeed, the NUPs showed a higher degree (degree > 17) than the other non-NUP proteins (p < 0.01, Fisher's exact test).

#### AM pharmacogenomics

**Figure 15:**
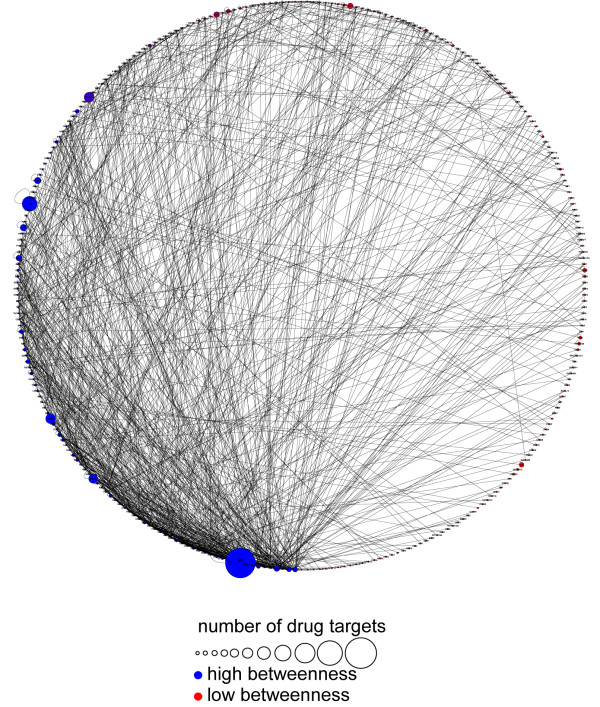
**Correlation between drug targeting and betweenness of nodes**. yFiles Circular Layout of AM network that emphasizes the nodes with high betweenness. The nodes with high betweenness are localized on the left half of the circle (blue colour and highest density of the edges). These proteins are characterized by their propensity to be drug targets, as shown with the major size of nodes.

By plotting the available data related to drugs targeted at the AM network, we found that most of AM proteins targeted by drugs were characterized by high connectivity; particularly, there was a highly significant association between the betweenness of these proteins and their being targets of drugs (p < = 0.004072, Wilcoxon Signed-Rank Test) (Figure 15).

#### Apoptosis induced by imatinib in K562 cells and by fenretinide in SH-SY5Y cells

**Figure 16:**
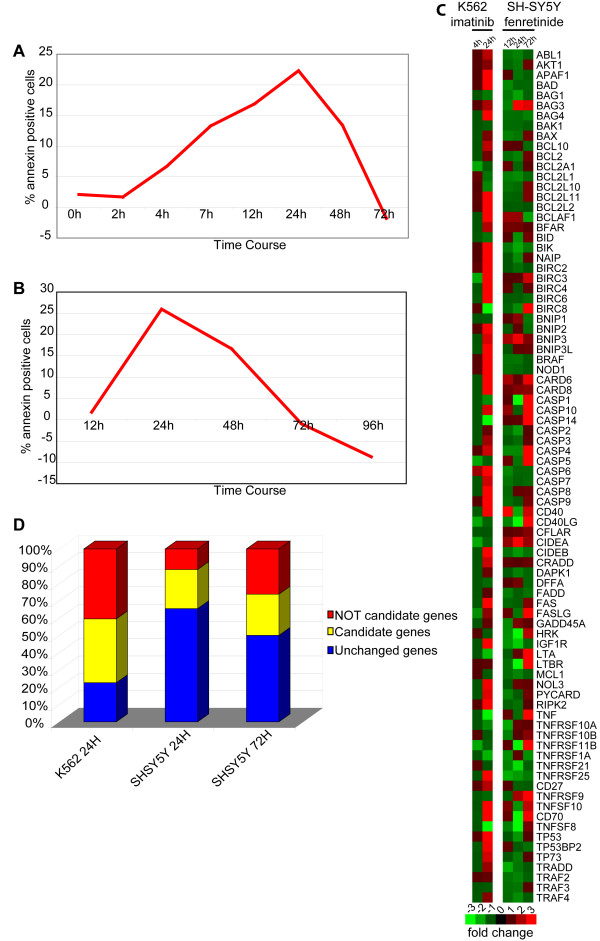
**Apoptosis induced by drugs**. Panel A: Kinetics and extent of apoptosis induction after exposure of K562 cells to 1 μM Imatinib (as determined through the annexin test and FACS analysis). Panel B: Kinetics and extent of apoptosis induction after exposure of SH-SY5Y cells to 3 μM of Fenretinide. Panel C: Modification of the AM transcriptome in K562 and SH-SY5Y after drug treatment (see Materials and Methods). Panel D: Modification of the expression of AM cancer candidates (i.e., previously identified as potentially involved in the pathogenesis of specific cancers) as opposed to that of other AM genes, presumed not to be involved in the process (i.e., not candidates) during apoptosis induction in K562 and SH-SY5Y.

A time course FACS analysis with Annexin V-FITC demonstrated that following treatment of K562 cells with Imatinib 1 μM, annexin-positive cells appeared 12 h after treatment (17% of treated cells). The percentage of apoptotic cells reached its peak 24 h after treatment (22.3%), while the conclusion of the apoptotic process apparently occurred within 72 h (Figure 16, Panel A). Real time PCR transcriptome analysis of 84 AM core genes was performed at 4 h after induction (before K562 cells had shown any phenotypic sign of apoptosis) and at 24 h (at the peak of apoptosis according to annexin positivity) (Figure 16, Panel A). When checked with the 2^-ΔΔ*CT *^method, the 4 h point showed no significant change of AM genes expression with respect to the control, while the same analysis at the 24 h point revealed remarkable modifications in the transcriptome: these consisted of an up regulation of the genes that trigger or positively control the apoptotic mitochondrial pathway (proapoptotic members of the BCL2 family, inhibitors of BCL2 and BCL-XL, apoptosome members), caspases (CASP3, CASP4, CASP6, CASP7, CASP8, and CASP9), or their positive regulators. More specifically, the following pro-apoptotic AM genes reached a peak of expression at this time point: APAF1, BCLAF1, BIK, TNFSF10, and TNFRSF25 (Figure 16, Panel C). TP73 was over expressed with respect to the control, suggesting a possible activation of a DNA repair pathway or the activation of genes that positively control apoptosis or negatively modulate the cell cycle. The increased expression of many caspases is concomitant to the up regulation of BIRC family members (caspases inhibitors), suggesting a co-existing subpopulation of cells resistant to apoptosis induction (Figure 16, Panel C). Focusing on the specific biological pathways activated in this model, we found that most of the AM genes down regulated at 24 h belong to the TNF/Stress Related Signaling and SODD/TNFR1 Signaling Pathways, while the members of the caspase cascade and mitochondrial apoptotic signalling were all up regulated. Among the AM genes whose expression was apparently unaffected in our model, about 70% are potential candidates for involvement in leukemia (Figure 16, Panel D). The Annexin-V propidium test on SH-SY5Y, treated with fenretinide 3 μM, showed that apoptosis peaked 24 h after drug administration and was apparently concluded at 72 h (Figure 16, Panel B). A real time PCR analysis of AM *cor*e genes was performed in the early phases of treatment (12 h), at the point with the highest percentage of annexin positive cells (26%) (24 h), and at the conclusion of the process (72 h). Expression of pro-apoptotic genes GADD45A, TNFRSF1A, and TNFRSF10A peaked at 24 h. Similar to K562 cells, the real-time expression data in treated SH-SY5Y cells showed a massive activation at 72 h of the caspase family genes CASP1, CASP4, CASP5, CASP10, and CASP14, including CASP8, that is emi-methylated in the SH-SY5Y cell line and has been reported to be induced after fenretinide treatment [77] (Figure 16, Panel C). 72 h after treatment both pro- and anti-apoptotic members of the Bcl2 family were induced: some pro-apoptotic members (i.e., BAX, BCL2L10, BCL2L11) began to increase slightly starting at 24 h, while some anti-apoptotic genes (i.e., BCL2, BCL2A1, MCL1.) were down regulated at 24 h. Death receptors and their ligands (CD40, CD40LG, CD70, FAS, FASLG, LTBR, TNF, TNFRSF11B, TNFRSF21, TNFSF8, TNFSF10) decreased during the post-treatment interval 12 h – 24 h, reaching their highest expression at 72 h; expression of the transducers AKT1 and RIPK2 followed a similar kinetics. Interestingly, the caspase inhibitors BIRCs, in particular BIRC8, showed an increased expression at 72 h. TP53 and TP73 expression values were lower at 24 h than at 12 h and increased at 72 h, while GADD45A exhibited its peak of expression at 24 h (Figure 16, Panel C). By analyzing the modulation of the biological pathways, we found that during the interval 12 h – 24 h many genes involved in the Caspase cascade and the TNF pathways were transcriptionally down regulated, while during the interval 24 h – 72 h most of genes up regulated were members of the Fas and TNF pathways, the Caspase cascade and the mitochondrial apoptotic signalling. The number of core AM genes, transcriptionally changed after Fenretinide administration, increased during the time course (about 34% and 50% at 24 h and 72 h, respectively) (Figure 16, Panel D). Similar to K562 cells, an important fraction of transcriptionally unaltered genes (about 60%) are candidates, previously identified for their potential involvement in neuroblastoma: as with the K562 cell line, this may suggest that their activation is prevented by critical mutations. Again similar to K562 cells, we also detected a modification of the expression of AM genes that based on our computational analysis were not expected to be involved in neuroblastoma pathogenesis: this confirms, if needed, the importance of the experimental verification of computational data (Figure 16, Panel D).

## Discussion

### The omic characterization of the apoptotic machinery: a model for a system biology approach to *Homo sapiens *biopathology

Understanding the molecular bases of complex and biomedically important phenotypes, as cancer and degenerative diseases, is one of the critical challenges for contemporary BioMedicine [14,78]. Different approaches have been proposed to explain cancer onset and heterogeneity, ranging from the study of single genes to large-scale system analysis [79,80]. The molecular oncology data obtained in the last decades have shown that the neoplastic phenotype is mainly due to genetic or epigenetic defects of two complex molecular machineries: the Cell Cycle and DNA Replication Apparatus (CCDRA) and the Apoptotic Machinery (AM) [22,82,83]. Typically, the biopathology of tumour cells is double faced: these cells are prone to uncontrolled proliferation with a scarce propensity to differentiate [84,85], but they also possess an ever evolving tendency to escape cell death: the consequent *immortality *provides them with a strong selective advantage over the wild-type ones, also endowing them with resistance to chemotherapy. Accordingly, many mutations and dysregulations of CCDRA and AM genes are found in all cancer types [22,82,83]. However, the involvement of apoptosis dysfunctions in cancer has remained elusive at the system level. Based on these premises, we decided to characterize the *Omics *of the Apoptotic Machinery for using them: (i) as a tool to identify new cancer genes; (ii) to profile cancers for diagnostic purposes; and finally, (iii) to pinpoint new targets for pharmacological therapy. Our data suggest that the potential involvement of AM genes is heterogeneous in different cancer models, both for number and type of chromosomal breakpoints as for frequency of genomic mutations, suggesting heterogeneity of pathogenesis (Figures 5, 6). The highest levels of chromosomal mutations involving AM loci were observed in neuroblastoma, lung and pancreatic cancers (Figure 6). Their paucity in other cancers suggests either different pathomolecular mechanisms (e.g., still uncharacterized point mutations outside coding regions), or the involvement of other cellular machineries and pathways, such as CCDRA or the DRA [86,87].

### AM structure and evolution

Few AM peripheral proteins are expressed in Prokaryotes and unicellular Eukaryotes, possibly with biological roles unrelated to metazoan apoptosis [10]. At the time of pluricellularity onset, the molecular *core *of apoptosis inductors and executors (caspases, death receptors, and adapters) was already assembled (Figure 1). With the appearance of Vertebrates, the evolution of the Apoptotic Machinery proceeded by *accretion *[88,89] (that is, through the recruitment of new molecules capable of interacting with the existing ones), possibly accelerated by the presence of molecular motifs favouring specific interactions. Its complexity further increased with the arrival of mammals, very likely due to the expansion of gene families that characterized their evolution (Figure 1) [90-92]. This is a common trend in the evolution of Eukaryotes, previously observed for other molecular machineries such as those controlling intracellular molecular trafficking or RNA interference [93,94]. The analysis of a more complex molecular apparatus (such as CCDRA) showed that *Homo sapiens *shares only 60% of its CCDRA genes with other eukaryotes (e.g., *Saccharomyces cerevisiae *and *Arabidopsis thaliana*). Interestingly, a sizeable fraction of the genes expressed during the cell cycle do not have orthologs in all the organisms analyzed: this suggests that each species may also synthesize a different and specific set of cell cycle proteins [95,96]. By analogy, the presence of different AM transcriptome profiles in different organisms could be expected. Phylogenetic analysis of unicellular eukaryotes identified orthologs of several AM genes encoding transcription factors and kinases, that in Metazoa are involved in the control of cell cycle, proliferation, differentiation and apoptosis (Figure 1). This could suggest that a molecular apparatus, responsible for the execution of a genetically guided form of cell death, was present in the first Eukaryotes: however, many of the genes performing it seem to be exclusive of unicellular eukaryotes [97]. As expected, higher Eukaryotes share only few AM genes with Bacteria. It seems logical to hypothesize that the biomolecular function of these proteins could be different in the two Domains and not necessarily bear a functional relationship with cell death in Bacteria (*original sin *hypothesis) [10,98]. AM genes shared with viral genomes (both DNA and RNA viruses) are transcription factors involved in the control of cell cycle, proliferation, differentiation and in the MAP kinase pathway. These viral genes possess an oncogenic potential and presumably are the result of a horizontal gene transfer from vertebrates [99]. Most AM proteins show a medium-to-low conservation degree and have an evolutionary rate similar to that of fibrinopeptide (Figure 1). This high rate of evolution suggests a relaxed structure/function relationship, that would allow them to stand many types of mutations without impairing their biological role. Many AM genes were found to be mutated in different types of neoplasia. Indeed, the loss of the self-destruction ability of a cell gives it an immediate selective advantage over the others, even though it may be detrimental for the whole organism [100]. Furthermore, almost 50% of AM genes are members of a gene family: these genes tend to evolve more quickly because of the functional redundancy that characterizes the family after their duplication [101,102]. AM proteins characterized by a medium-to-high conservation degree (< 10%) are peripheral AM nodes, frequently at the boundaries with other cell machineries: they also are involved in other important functions, as cell cycle regulation (e.g., AKT3, the members of the cullin family, GSK3B, and SRC), signal transduction (e.g., HRAS, MAPK, PDCD6, YWHAE, YWHAG), metabolism, post-translational modifications (e.g., BECN1, MASK, RPL5, STK25) (Figure 1). Their biological role mainly consists in the regulation of the activity of receptors, transductors, and executors of the apoptotic program. Since they perform a critical role, these molecules were subjected to a strong selective pressure and have largely preserved structure and functions. In many cases, these proteins function within some of the most ancient biological processes: about 50% of these molecules are found in unicellular Eukaryotes and in Prokaryotes (e.g., the MAP kinases) [103]. Our data confirm results previously published on the evolution of the human genome: proteins operating in regulatory processes (e.g., transcription factors and receptors) have remained relatively well conserved during eukaryotic evolutions [104]. However, it remains true that the most conserved genes are those involved in such critical biological processes as metabolism, protein synthesis, and molecular transport [104]. Our data seem also to suggest that in most of cancer models the genome regions most frequently affected by loss-type mutations contain the most conserved AM genes, suggesting a strong phenotype – genotype correlation (see Results, Oncogenomics).

### Specificity of the AM transcriptome: cancer profiling

The analysis of the AM transcriptome profile showed that the transcriptional involvement of this apparatus is heterogeneous in different cancer models for number and type of AM genes involved. These data suggest that AM could be involved in different tumours through tumour-specific mechanisms of transcriptional activation or repression [105,106]. According to our data, the main sources of AM transcriptional failure are represented by apoptosis regulators (i.e., transcription factors, kinases, and death receptors) rather than by its executors (e.g., members of the apoptosome, death transductors, and caspases) (Figure 7). This could suggest that frequently exploited mechanisms for cancer transformation are mutations of the genes at the top of the death signalling network: they are generally at the boundaries with other molecular machineries involved in cancer, such as the CCDRA or the DRM, and could have a pleiotropic effect on the activities of several other genes (Figure 7). The identification of specific AM molecular signatures (cancer profiling) could be useful for precise tumour diagnosis and specific therapy design, distinguishing the different molecular alterations associated to different tissues. As the number of AM genes transcriptionally altered in cancers is reasonably limited (between 25 and 200), specific low-cost platforms for AM transcriptome analysis could be designed for routine molecular screening of patients to understand the origin of the cancer and its metastases [107,108]. Furthermore, the comparison of molecular profiles would allow: (i) the identification of the common alterations among tumours; (ii) to understand the common basis of AM involvement in cancer transformation [107,108]. It is notable that a fraction of AM transcriptome dysregulations could be the result of an altered control of expression of these genes, triggered by the metabolism and cell cycle imbalance, and characteristic of neoplastic cells; in some cases, they could be due to aberrant gene methylation [83]. Various transcriptome alterations were mirrored in the proteome and in some cases we also detected the corresponding genomic mutations (Figure 12). This approach allowed us to obtain a list of very reliable candidates for the malfunctioning apoptosis in cancer cells (Tables 7, 8). It is interesting to note that our functional analysis on AM dysregulated genes and proteins, based on GO, showed a possible different regulation of anti-apoptotic and pro-apoptotic genes. This could have different explanations: (i) its molecular basis could be a qualitative and quantitative mismatch between transcriptome and proteome, possibly due to post-transcriptional modifications, the latter being the real cause of cell phenotype [109,110]; (ii) the apoptotic cascade is mainly a protein signalling network based on protein/protein interaction and PTM (post-translational modification), frequently proteolysis and phosphorylation: usually, cells have most of the apoptotic actors constitutively present but in the inactive state [7]; (iii) blocking of apoptosis does not necessarily require a global down-regulation of all pro-apoptotic genes or an overall up-regulation of all anti-apoptotic genes: in principle, it is sufficient that the expression of only a few of these genes (logically the hubs of the AM network, acting as masters) were altered to functionally modify cell behaviour [111]. We should expect that the relationship between AM qualitative alterations and the neoplastic phenotype is neither consistent nor simple to read [112].

### AM network and cancer therapy

The mere qualitative and quantitative analysis of the alterations of a molecular machinery could be misleading, if not inserted in a tetradimensional *scenario *(the three dimensions of space plus time) that would allow one to detect the relevance and the functional influence of all the elements of the machinery within the organism. The AM network shows a very interesting structure. Namely, approximately 50% of its core proteins (effectors and executor of death) have at least one death domain (DEATH, DED, CARD, and DAPIN): this demonstrates a very efficient strategy of modular signal transduction (Figures 2, 4). Only a few modules are sufficient to establish the reciprocal interactions among at least 50 different proteins. The relatively small number of functionally related protein modules within AM suggests a possible evolutionary pathway. Most likely, when the molecular machinery responsible for apoptosis first appeared, it may have comprised a small group of proteins, characterized by few classes of motifs and domains, which allowed reciprocal protein interactions to induce, transduct and execute the apoptotic process. The subsequent gene duplication events and the phenomena of exon shuffling or meiotic recombination determined the evolution of the apparatus. This phase included its growth by accretion due to the recruitment of new interacting partners, spurred by newly acquired molecular sequences important for specific molecular interactions [88,89]. However, the structural and functional protein modules remained unchanged, together with the signals modulating and executing apoptosis [113,114]. Some AM proteins are NUPs: this suggests that for an appropriate control of apoptosis it may be useful that some proteins possess a malleable structure, endowing them with functional features not shown by other rigidly ordered proteins involved in death signalling [115,116]. The advantages of the great conformational freedom of NUPs are the ability to fold upon binding to their biological targets (coupled folding and binding) and an increased speed of interaction. Their extended structure and freedom in orientational search enable them to contact their partners over a large binding surface and to recognize distant determinants on the target [115,116]. This flexibility may be critical for the assembly of specific macromolecular complexes, which cannot be made of rigid components because of their sizes and topological constraints. Finally, the NUPs have an extreme proteolytic sensitivity that allows them to very rapidly turn over upon receiving appropriate signals. We found that NUPs typically represent the hubs of the AM network, suggesting that their structural plasticity makes them very important for the biology of the cell (Figure 6). Malfunctions of these dynamic nodes affect the stability of the network and seriously modify cell behaviour. It is not surprising that many cancer-related AM genes carrying point mutations are NUPs, in particular some of those frequently mutated (as HRAS, TP53, and CDKN2A) (Figure 6). The analysis of AM alterations related to cancer, based on a network view, has pinpointed the critical role of the hubs, the structurally and functionally most important nodes of the network, which could critically impair network's stability and accordingly the physiologic status of the cell [117]. In fact, these nodes are lethal – embryonic perinatal or lethal postnatal genes in knock-out mice, and unsurprisingly they show genomic alterations, coupled to transcriptional dysregulations, in many cancer models [118,119]. Moreover, we found that highly connected nodes are frequently mutated (Table 3; Figures 3, 6). All these data seem to suggest that tumour-related defects, occurring in the hubs of AM, are preferentially selected. The functional impairment of a few nodes, which control directly or indirectly the activities of many others in the context of the co-occurrence of multiple genetic defects, could represent a selective advantage during neoplastic transformation (Additional file 12). The relationship between network connectivity and molecular alterations of AM is also stressed by our observation that the genes with more links to mutated genes are themselves highly susceptible to be dysregulated in cancer (Figure 14). We propose to exploit this approach, based on the search for nodes connected to AM mutated hubs, to discover new cancer biomarkers [22]. Accordingly, the knowledge of the critical role of AM hubs in the neoplastic phenotype could provide us with a powerful strategy for designing targeted pharmacological treatment. Apoptosis induction as strategy for cancer treatment is one of the most exploited therapeutic approaches. As we have shown in this paper, activation of cell death is the common result of the administration of different drugs (e.g., Imatinib and Fenretinide) in different cancer types (e.g., CML and neuroblastoma). Imatinib and Fenretinide work by triggering different AM pathways of molecular signalling (by blocking the ATP binding site of BCR-ABL or by highly increasing the production of ROS, respectively). However, these biochemical cascades lead to the same final event: apoptotic death [120,121]. The real – time transcriptome analysis of K562 and SH-SY5Y cells (treated with Imatinib and Fenretinide, respectively) showed similar expression trends, although with a different correlation to the corresponding morphological events (Figure 16). This analysis agrees with the observation that their AM dysregulation profiles at steady-state are very similar (Figure 7, Panel C). Caspase activation, coupled to the triggering of mitochondrial death and partial suppression of the extrinsic death pathway, are the common changes observed in both cancer cell lines following pharmacological administration. The transcriptome changes observed during apoptosis induction involved both AM genes dysregulated in cancer as AM genes previously thought to be unaffected (Additional file 10). We note that the expression of some AM genes, previously identified as candidates for leukaemia and neuroblastoma, was apparently unchanged in our models: this behaviour may suggest they may have suffered functional impairment, due to genomic mutations or epigenetic modifications. The choice of the molecular cascade, leading to drug induced – apoptosis, is probably constrained by defects in some parts of the death machinery (e.g., mutations or epigenetic repression of death receptors). Therefore, the knowledge of the cancer-associated alterations of AM could be a critical tool to predict whether a pharmacological treatment, based on apoptosis induction, will be effective [20,122]. The proposal to identify all of the weak points along the AM network of specific cancers could be exploited for designing pathway-specific therapies to induce apoptosis in tumours. This strategy could be especially useful for those cancers that apparently are very resistant to death induction: the exact knowledge of their AM's Achilles' heels should allow the identification of alternative molecular targets, usable to induce apoptosis by new unaffected and unexplored pathways [123]. As the hubs seem to be the major actors in apoptosis defects related to cancer, they could be considered very appropriate targets for drug design. In fact, we are not surprised that most of AM proteins, targeted by drugs, are characterized by a high degree of "betweenness" (Figure 15). These characteristics indicate their relevance as organizing regulators within a specific network and their potential influence on maintaining the functions of signalling mechanisms [124]. Ideally, drugs should be selected so as to preferentially target proteins controlling complex signalling cascades, rather than functionally isolated nodes: consequently, they would affect the behaviour of the cell in a significant way to produce relevant clinical effects (Figure 15). Moreover, the pharmacological targeting of the critical nodes of the AM network should be extended also to small RNAs that participate in regulating apoptosis. Our data suggest that more than 100 MIRs potentially target AM genes. These MIRs are localized in frequently altered regions in cancer and have an altered expression in many tumours (Figures 5, 9; Tables 5, 6). As already reported [125,126], MIRs have an important role in the pathogenesis of cancer and it may be expected that many of them could have a critical function in determining defects of AM functions [127,128]. Knowledge of dysregulated MIRs and their target specificity could be used for new therapeutic approaches based on blocking the up regulated MIRs (e.g., by using the antagomirs) or restoring their function (by MIR transfection with liposoluble or synthetic carriers, such as microspheres) [129,130]. The use of MIRs as therapeutic repressors of oncogenes would be an innovative medical strategy, which could improve the efficacy of traditional pharmacological therapies, or in some cases replace it [131].

## Conclusion

We suggest that the Omic characterization of the Apoptotic Machinery, a critically important biological apparatus, could pave the way for further studies and critical applications in the world of Molecular BioMedicine with important perspectives both for Oncology as well as for Regenerative Medicine and Stem Cell Biology (D Barbagallo, S Piro, M Ragusa et al., 2009, submitted). We are encouraged to believe that this approach, if also applied to other cell machineries as CCDRA and DRA, will eventually allow the identification of the molecular signals critically involved in not just the normal functioning of an organism, but also in its differentiation from the zygote. Time could now be ripe for the emergence of a new research field, that under the name of Signal Biology could focus on the search for the determinants of specific molecular interactions within organisms and their cells. Such a field would rely on the vast amount of biomolecular data already available and amenable to manipulation by the wide set of tools devised by the Computational Biology and Experimental BioMedicine communities. Data from these integrated studies should be critically important to deepen our knowledge of development and differentiation, as well as of biological complexity and its relationship to the phenotype. They should also help to intelligently devise strategies for therapeutic interventions and individualized Medicine, and eventually for fulfilling the promises of Synthetic Biology and Bionics [132,133].

## Abbreviations

(AM): Apoptotic Machinery; (CCDRA): Cell Cycle and DNA Replication Apparatus; (CML): Chronic Myelogenous Leukemia; (DRA): DNA Repair Apparatus; (GO): Gene Ontology; (HT): High Throughput; (MIRs): MicroRNAs; (NUPs): Natively Unfolded Proteins; (PTMs): Post-Translational Modifications.

## Competing interests

The authors declare that they have no competing interests.

## Authors' contributions

MP conceived and coordinated the project; MP, CD, MR, SL, SS, FD, BM designed experiments, the other Researchers performed them; all contributed to the critical revision of the data; MP, CD, MR, BM wrote the paper. All Authors contributed to its revision.

## Pre-publication history

The pre-publication history for this paper can be accessed here:



## Supplementary Material

Additional file 1 **Gene symbol, name and localization of AM protein-coding genes and AM MIR-coding genes** Click here for file

Additional file 2 **GO classification of AM genes**. Panel A. General Function of AM protein – encoding genes. Panel B. Biological Processes. Panel C. Molecular Functions. Panel D. Subcellular Localization. Panel E. General Function of AM genes targets of MIRs. Click here for file

Additional file 3 **Experimentally verified MIRs targeting AM genes.** Click here for file

Additional file 4 **MIR host genes in AM.** Click here for file

Additional file 5 **AM genomics**. Panel A. Genome Map of AM Genes. Panel B. Comparison of the genome distribution of AM genes with respect to the other human genes. Click here for file

Additional file 6 **Genome clusters of AM genes in human, chimpanzee, mouse.** Click here for file

Additional file 7 **Proteomic features of AM**. Panel A. Molecular weight distribution of 548 AM proteins. Panel B. Percentage of post-translational modifications of AM proteins. Panel C. Percentage of metal ions within the tertiary structure of AM proteins. Click here for file

Additional file 8 **Distribution of protein motifs and domains in AM.** Click here for file

Additional file 9 **AM genes positional candidates in cancers (data from array-CGH).** Click here for file

Additional file 10 **AM transcriptome data.** Click here for file

Additional file 11 **AM genes localized in loss- or gain-regions and down or up regulated, respectively.** Click here for file

Additional file 12 **Features of AM hubs.** Click here for file
