# Supplementary figures and images for: The apoptotic machinery as a biological complex system: analysis of its omics and evolution, identification of candidate genes for fourteen major types of cancer, and experimental validation in CML and neuroblastoma
Source: BMC Med Genomics. 2009 Apr 30;2:20. doi: 10.1186/1755-8794-2-20 (PMC2683874; doi:10.1186/1755-8794-2-20)

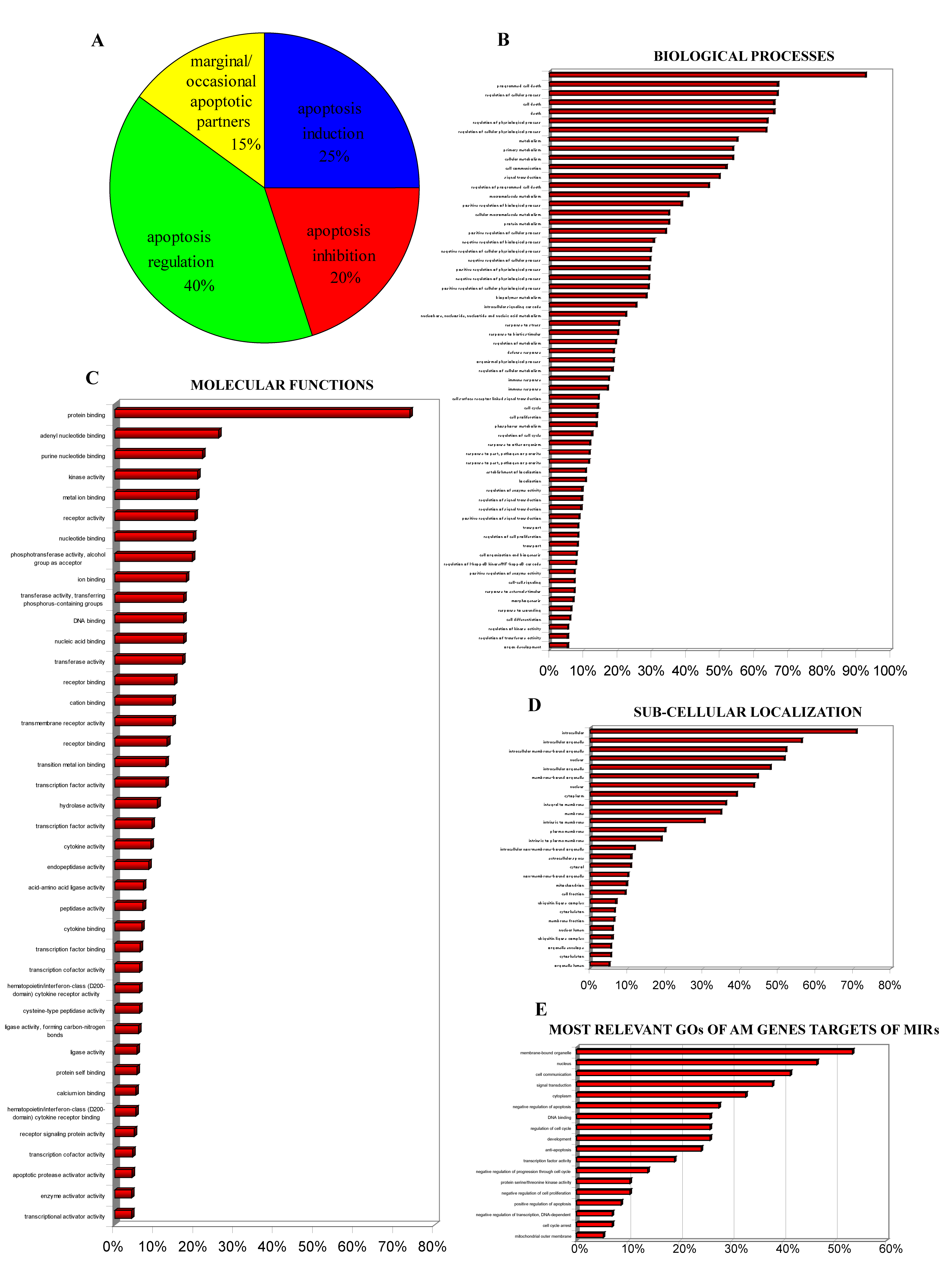

Supplement: Additional file 2 — GO classification of AM genes. Panel A. General Function of AM protein – encoding genes. Panel B. Biological Processes. Panel C. Molecular Functions. Panel D. Subcellular Localization. Panel E. General Function of AM genes targets of MIRs. [file 1755-8794-2-20-S2.tiff]

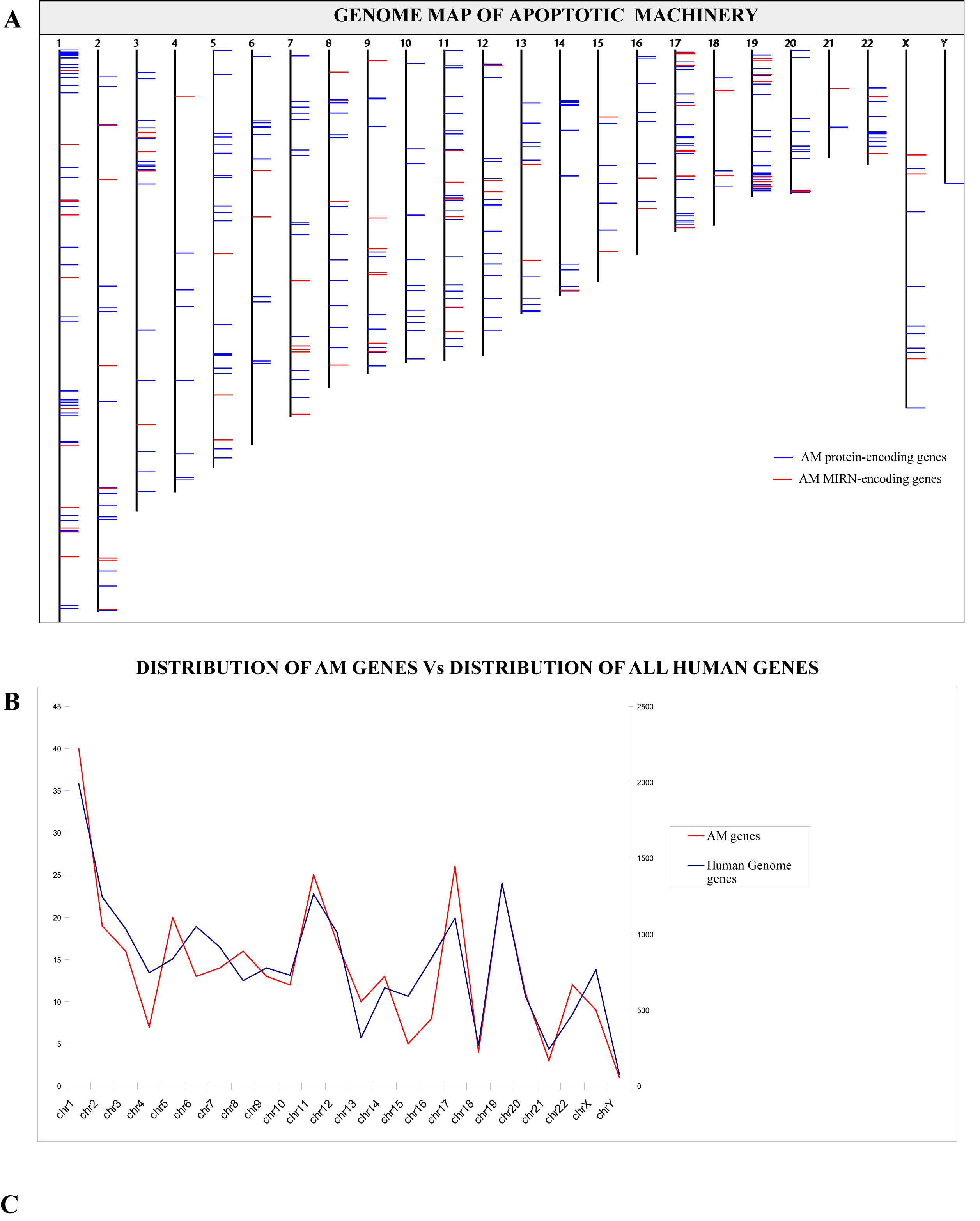

Supplement: Additional file 5 — AM genomics. Panel A. Genome Map of AM Genes. Panel B. Comparison of the genome distribution of AM genes with respect to the other human genes. [file 1755-8794-2-20-S5.tiff]

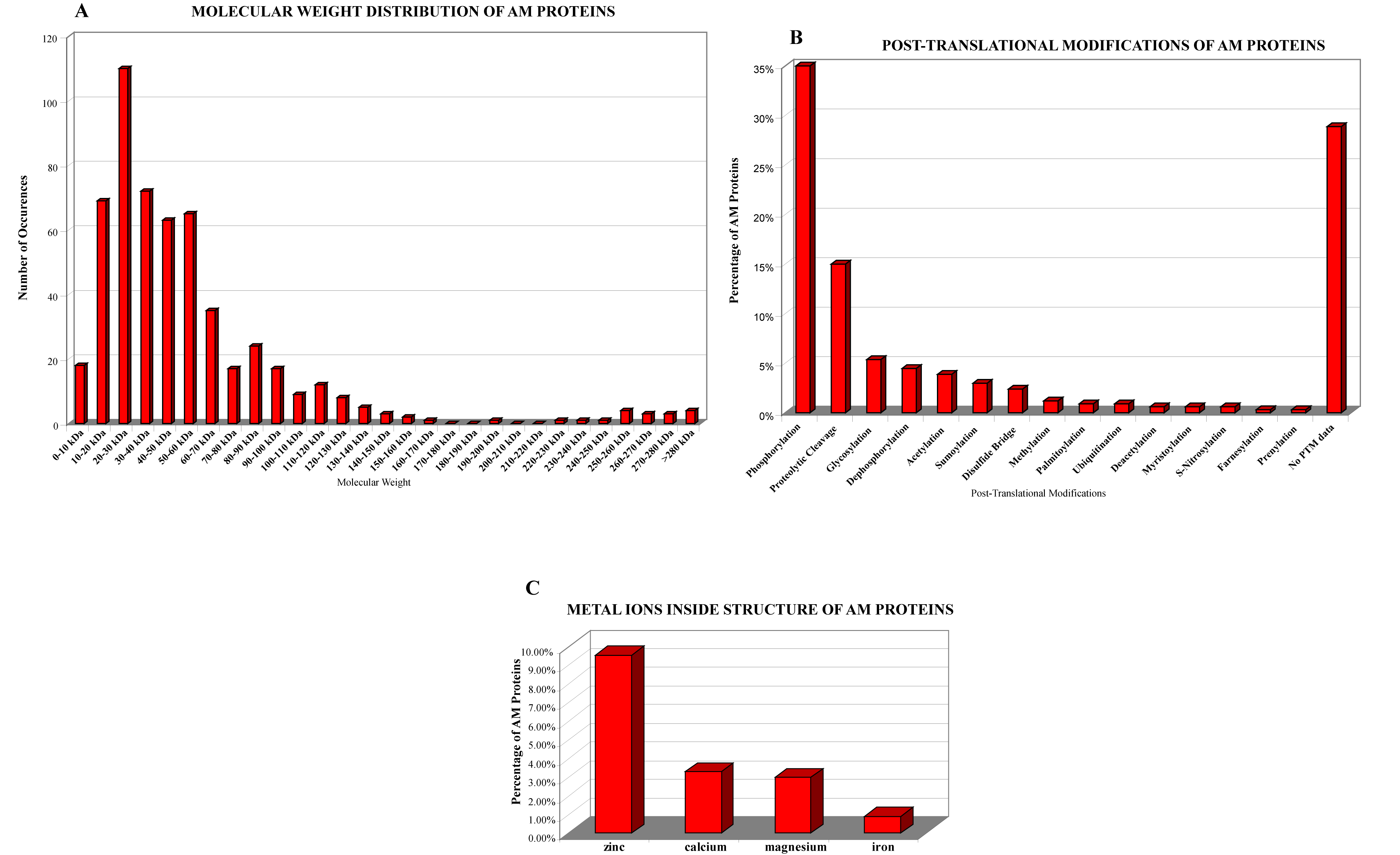

Supplement: Additional file 7 — Proteomic features of AM. Panel A. Molecular weight distribution of 548 AM proteins. Panel B. Percentage of post-translational modifications of AM proteins. Panel C. Percentage of metal ions within the tertiary structure of AM proteins. [file 1755-8794-2-20-S7.tiff]

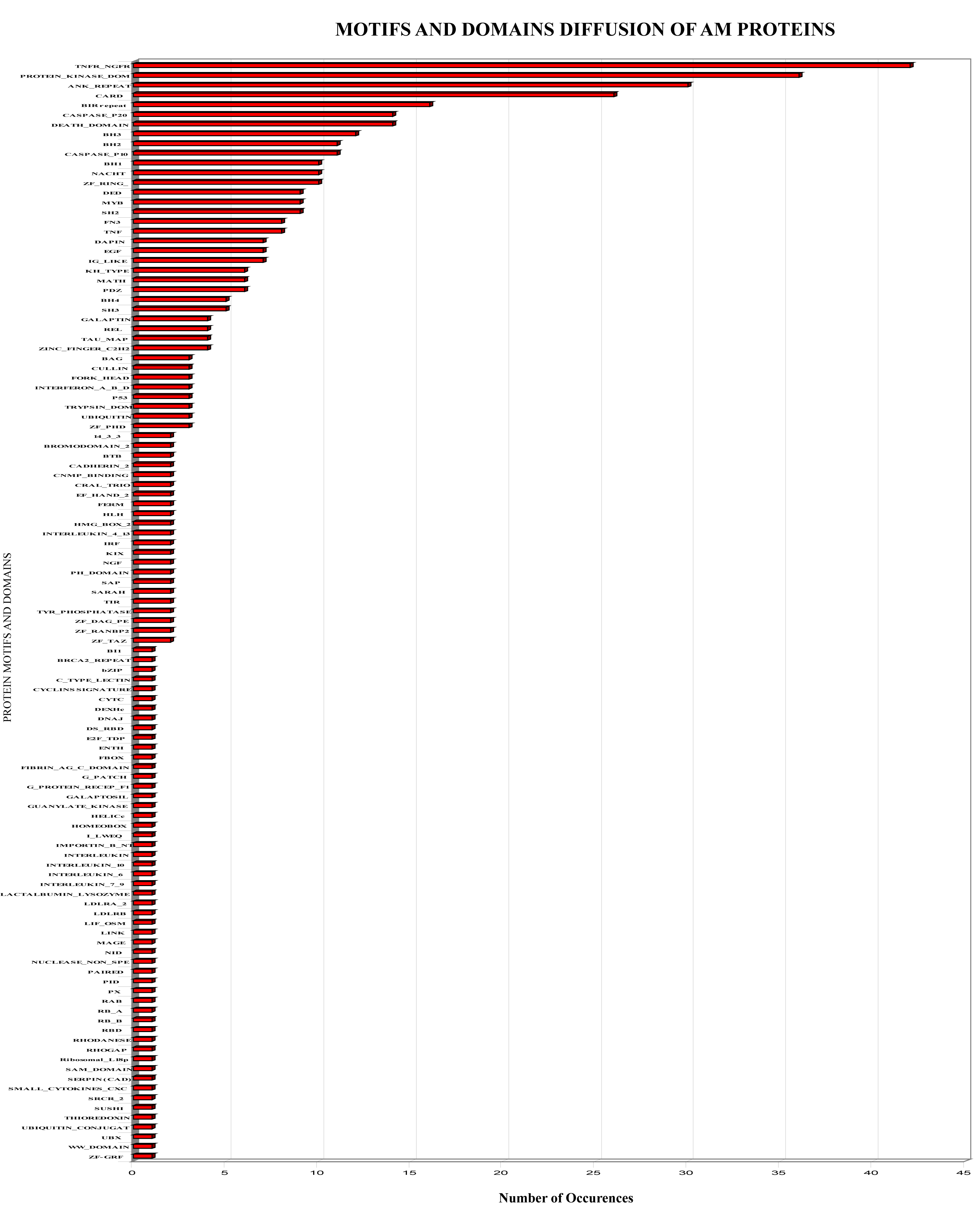

Supplement: Additional file 8 — Distribution of protein motifs and domains in AM. [file 1755-8794-2-20-S8.tiff]
