# Supplementary material for: The apoptotic machinery as a biological complex system: analysis of its omics and evolution, identification of candidate genes for fourteen major types of cancer, and experimental validation in CML and neuroblastoma
Source: BMC Med Genomics. 2009 Apr 30;2:20. doi: 10.1186/1755-8794-2-20 (PMC2683874; doi:10.1186/1755-8794-2-20)
Supplement: Additional file 3 — Experimentally verified MIRs targeting AM genes. [file 1755-8794-2-20-S3.doc]

**Experimentally Verified MIRs Targeting AM Genes**

| **AM GENES** | **MIRNs** | **REFERENCES** |
| --- | --- | --- |
| **B4GALT1** | MIR124 | Lim et al., Nature (2005) |
| **BCL2** | MIR15, MIR16 | Croce et al., Proc Natl Acad Sci U S A (2005) |
| **BDNF** | MIR1b | Lewis BP et al., Cell (2003) |
| **DFFB** | MIR124 | Lim et al., Nature (2005) |
| **HTATIP2** | MIR124 | Lim et al., Nature (2005) |
| **MYBL1** | MIR373 | Lim et al., Nature (2005) |
| **MYCN** | MIR101 | Lewis BP et al., Cell. (2003) |
| **PDCD4** | MIR1 | Lim et al., Nature (2005) |
| **RB1** | MIR106a | Volinia et al., Proc Natl Acad Sci U S A. (2006) |
| **RELA** | MIR124, MIR373 | Lim et al., Nature (2005) |
| **STK4** | MIR373 | Lim et al., Nature (2005) |
| **TGFBR2** | MIR20a | Volinia et al., Proc Natl Acad Sci U S A (2006) |
| **TNFRSF21** | MIR124 | Lim et al., Nature (2005) |
| **TUSC2** | MIR373 | Lim et al., Nature (2005) |
