# Supplementary material for: The apoptotic machinery as a biological complex system: analysis of its omics and evolution, identification of candidate genes for fourteen major types of cancer, and experimental validation in CML and neuroblastoma
Source: BMC Med Genomics. 2009 Apr 30;2:20. doi: 10.1186/1755-8794-2-20 (PMC2683874; doi:10.1186/1755-8794-2-20)
Supplement: Additional file 4 — MIR host genes in AM. [file 1755-8794-2-20-S4.doc]

MIR Host Genes In AM

|  |  |
| --- | --- |
| **AM genes** | **host** |
|
| **AATK** | MIR657, MIR338 (intron 7) |
| **BIRC6** | MIR558 (intron 61) |
| **DAPK3** | MIR637 (intron 5) |
| **GULP1** | MIR561 ( 5’UTR ) |
