# Supplementary material for: The apoptotic machinery as a biological complex system: analysis of its omics and evolution, identification of candidate genes for fourteen major types of cancer, and experimental validation in CML and neuroblastoma
Source: BMC Med Genomics. 2009 Apr 30;2:20. doi: 10.1186/1755-8794-2-20 (PMC2683874; doi:10.1186/1755-8794-2-20)
Supplement: Additional file 6 — Genome clusters of AM genes in human, chimpanzee, mouse. [file 1755-8794-2-20-S6.doc]

Genome Clusters Of AM Genes In Human, Chimpanzee, Mouse

| **Cluster** | **AM genes** | **Human** | **Chimp** | **Mouse** |
| --- | --- | --- | --- | --- |
|  |  |  |  |  |
| **1p36** | *DFFA* | conserved | conserved | conserved |
|  | *DFFB* | conserved | conserved | conserved |
|  | *TNFRSF18* | conserved | conserved | conserved |
|  | *TNFRSF14* | conserved | conserved | conserved |
|  | *TNFRSF25* | conserved | conserved | conserved |
|  | *TNFRSF9* | conserved | conserved | conserved |
|  | *TNFRSF8* | conserved | conserved | conserved |
|  |  |  |  |  |
| **9p21** | *IFNB1* | conserved | conserved | conserved |
|  | *IFNA2* | conserved | conserved | conserved |
|  | *IFNA1* | conserved | conserved | conserved |
|  |  |  |  |  |
| **11q22** | *BIRC3* | conserved | conserved | conserved |
|  | *BIRC2* | conserved | conserved | conserved |
|  | *CASP4* | conserved | conserved | conserved |
|  | *CASP5* | conserved | conserved | lacking |
|  | *CASP1* | conserved | conserved | conserved |
|  | *CASP12* | pseudogene | functional gene | functional gene |
|  |  |  |  |  |
| **17q21** | *STAT5B* | conserved | conserved | conserved |
|  | *STAT5A* | conserved | conserved | conserved |
|  | *STAT3* | conserved | conserved | conserved |
|  |  |  |  |  |
| **19q13** | *NALP12* | conserved | conserved | conserved |
|  | *NALP2* | conserved | conserved | conserved |
|  | *NALP9* | conserved | conserved | conserved |
|  | *NALP8* | conserved | conserved | lacking |
|  |  |  |  |  |
| **21q22** | *IFNAR1* | conserved | conserved | conserved |
|  | *IFNAR2* | conserved | conserved | conserved |
