# Supplementary material for: The apoptotic machinery as a biological complex system: analysis of its omics and evolution, identification of candidate genes for fourteen major types of cancer, and experimental validation in CML and neuroblastoma
Source: BMC Med Genomics. 2009 Apr 30;2:20. doi: 10.1186/1755-8794-2-20 (PMC2683874; doi:10.1186/1755-8794-2-20)
Supplement: Additional file 12 — Features of AM hubs. [file 1755-8794-2-20-S12.doc]

Features Of AM Hubs

| **HUB** | **CGH ARRAY** | **POINT MUTATION** | **METHYLATION** | **TRANSCRIPTOME** | **PROTEOME** | **MIRs** |
| --- | --- | --- | --- | --- | --- | --- |
| ABL1 |  | Leukemia, Lung, Skin | Leukemia | Up regulated in Colon, Lung; down regulated in Ovary |  |  |
| AKT1 | Loss in Colon, Neuroblastoma, Stomach |  |  | Up regulated in Leukemia, Pancreas; down regulated in Lung, Neuroblastoma | Down regulated in Ovary,Breast, Pancreas, Lung, Neuroblastoma |  |
| BCL2 | Gain in Neuroblastoma; loss in Colon, Pancreas |  | Breast, Colon, Prostate | Up regulated in Breast, Leukemia, Neuroblastoma, Pancreas; down regulated in Kidney | Up regulated in Lung, Leukemia, Neuroblastoma; down regulated in Thyroid. | MIR204, MIR211, MIR217, MIR34A |
| BCL2L1 | Loss in Breast, Liver, Lung, Ovary, Pancreas, Prostate |  |  | Up regulated in Breast, Leukemia; down regulated in Neuroblastoma | Down regulated in leukemia | MIRLET7A, MIRLET7B, MIRLET7C, MIRLET7G, MIR133A, MIR98 |
| CASP3 | Loss in Liver, Lung, Ovary, Neuroblastoma | Kidney |  | Down regulated in Breast, Ovary, Prostate; up regulated in Stomach | Up regulated in Breast |  |
| CASP8 |  | Skin | Liver, Neuroblastoma | Down regulated in Colon, Neuroblastoma; up regulated in Kidney, Leukemia, Lung | Up regulated in Colon; down regulated in kidney, Liver, Leukemia, Neuroblastoma |  |
| CASP9 | Loss in Colon, Neuroblastoma | Kidney |  | Up regulated in Kidney, Lung; down regulated in Neuroblastoma | Up regulated in Kidney; down regulated in colon, stomach. |  |
| CFLAR | Gain in Ovary |  | Neuroblastoma | Down regulated in Breast, Kidney, Leukemia, Neuroblastoma, Thyroid; up regulated in Ovary, Pancreas, Skin |  |  |
| CHUK | Loss in Neuroblastoma | Breast, Lung |  | Up regulated in Leukemia, Neuroblastoma | Up regulated neuroblastoma; down regulated breast | MIR145, MIR152, MIR26A, MIR26B,MIR30B, MIR30C, MIR34B |
| CREBBP | Gain in Breast |  |  | Up regulated in Breast, Colon, Lung, Ovary, Prostate; down regulated in Leukemia, Neuroblastoma | Up regulated in Liver, Pancreas, Stomach; down regulated in Breast, Lung, Ovary, Leukemia, Neuroblastoma | MIR137, MIR153, MIR26B |
| EP300 | Loss in Colon, Pancreas | Lung, Skin |  | Up regulated in Breast, Liver, Pancreas; down regulated in Leukemia, Lung | Up regulated in Ovary, Liver, Leukemia, Lung, Colon, Skin, Kidney, Prostate, Breast | MIR129, MIR152, MIR186 (down regulated in Kidney, Prostate and Lung), MIR200C (down regulated in Kidney), MIR217 |
| FADD | Loss in Neuroblastoma |  |  | Up regulated in Breast, Leukemia, Ovary, Prostate; down regulated in Neuroblastoma, Thyroid |  |  |
| JAK1 | Loss in Colon, Neuroblastoma | Stomach |  | Up regulated in Leukemia, Pancreas, Prostate, Stomach; down regulated in Neuroblastoma |  |  |
| MAPK1 | Loss in Breast, Colon |  |  | Up regulated in Breast, Leukemia, Liver, Prostate | Up regulated in Liver; down regulated, Lung, Colon, Thyroid, Breast, Kidney, Pancreas, Stomach |  |
| PIK3R1 | Loss in Lung, Ovary, |  |  | Up regulated in Breast, Colon, Ovary, Prostate | Up regulated in Ovary; down regulated in Colon, Lung, Stomach |  |
| PRKCZ | Loss in Colon, Kidney, Neuroblastoma, |  |  | Down regulated in Colon, Neuroblastoma; up regulated in Kidney, Leukemia, Skin | Up regulated in Ovary; down regulated in Lung, Kidney |  |
| RAF1 | Loss in Lung, Neuroblastoma, Pancreas | Lung, Ovary |  | Up regulated in Kidney, Skin; down regulated in Neuroblastoma, Stomach |  |  |
| RB1 | Loss in Colon, Liver, Lung, Prostate, | Breast, Colon, Kidney, Leukemia, Liver, Lung, Ovary, Prostate, Skin, Stomach | Breast, Leukemia, Liver, Lung, Neuroblastoma, Prostate, Skin, Stomach | Up regulated in Breast, Leukemia, Neuroblastoma | Down regulated in Lung, Colon, Skin, Stomach,Liver, Kidney, Leukemia, Prostate, Breast. | MIRLET7A, MIRLET7E, MIRLET7E, MIRLET7G, MIR7, MIR98 |
| SRC | Gain in Breast, Lung, Ovary, Pancreas, Stomach | Colon |  | Up regulated in Breast, Kidney, Leukemia, Neuroblastoma, Ovary, Pancreas, Skin, Stomach; down regulated in Liver, Prostate | Up regulated in Prostate, Pancreas, Stomach; down regulated Leukemia. |  |
| STAT1 | Gain in Ovary | Breast |  | Up regulated in Breast, Leukemia, Ovary, Pancreas, Prostate, Stomach; down regulated in Neuroblastoma | Up regulated in Breast, Colon, Pancreas, Stomach, Skin, Lung, Ovary, Kidney, Leukemia. |  |
| STAT3 | Gain in Breast, Liver, Neuroblastoma, Pancreas, Stomach |  |  | Up regulated in Leukemia, Pancreas, Prostate, Stomach; down regulated in Breast, Liver | Up regulated in Pancreas, Stomach, Skin,Colon,Ovary, Kidney,Liver,Leukemia; down regulated in Lung | MIR106B (down regulated in Kidney) |
| TNFRSF1A | Gain in Ovary | Skin |  | Down regulated in Breast, Prostate | Up regulated in Prostate, Stomach. |  |
| TP53 | Loss in Breast, Liver, Pancreas, Ovary, Stomach; gain in Neuroblastoma | Breast, Colon, Kidney, Leukemia, Liver, Lung, Neuroblastoma, Ovary, Pancreas, Prostate, Skin, Stomach | Breast, Liver | Up regulated in Breast, Leukemia, Neuroblastoma, Prostate | Up regulated in Breast, Stomach, Pancreas, Skin, Colon, Ovary; down regulated in Lung. |  |
| TRAF1 |  |  |  | Down regulated in Neuroblastoma | Down regulated in Kidney |  |
| TRAF2 |  | Skin |  | Up regulated in Breast, Leukemia, Lung, Neuroblastoma, Pancreas, Prostate, Skin | Up regulated in Liver, Kidney, Skin, Colon, Neuroblastoma; down regulated in Prostate, Stomach |  |
